# AF6 orchestrates macrophage polarization via JAK2-STAT3 signaling and supports intestinal regeneration by stimulating stem cell proliferation

**DOI:** 10.3389/fimmu.2026.1798313

**Published:** 2026-06-19

**Authors:** Tao Jian, Xiaoxia Dong, Jingwen Kong, Xinyu Wang, Meiyan Qi, Lixing Zhan, Lilei Zhuang

**Affiliations:** 1 Shanghai Institute of Nutrition and Health, Shanghai Institutes for Biological Sciences, University of Chinese Academy of Sciences, Chinese Academy of Sciences, Shanghai, China; 2 State Key Laboratory of Systems Medicine for Cancer, Shanghai Cancer Institute, Renji Hospital Affiliated to Shanghai Jiao Tong University School of Medicine, Shanghai, China; 3 Yiwu Central Hospital, the Affiliated Yiwu Hospital of Wenzhou Medical University, Yiwu, China

**Keywords:** AF6, IBD, macrophages, organoid, STAT3

## Abstract

**Introduction:**

Macrophages play a central role in regulating intestinal inflammation, resolution, and tissue repair. However, the upstream regulators that govern their polarization during colitis remain poorly defined.

**Methods:**

In this study, we used a dextran sulfate sodium (DSS)-induced colitis model to investigate the role of the scaffold protein AF6 in colitis pathogenesis.

**Results:**

We identify AF6 as a key regulator that promotes pro-inflammatory macrophage polarization and exacerbates colitis. Mechanistically, AF6 expression promoted JAK-STAT3 complex formation. In turn, its deletion in macrophages resulted in impaired JAK-STAT3 signaling pathway and a shift away from pro-inflammatory M1 polarization. Furthermore, the AF6-deficient macrophages were determined to also promote epithelial regeneration through enhanced IL-10 production and downstream activation of Wnt/β-catenin signaling in intestinal organoids culture.

**Discussion:**

Thus, our study defines a therapeutically amenable AF6-JAK2-STAT3 axis that controls macrophage-driven pathogenesis in colitis, revealing a novel strategy to break the cycle of inflammation and impaired repair.

## 1 Introduction

The intestinal epithelium undergoes a self-renewal process every 4–7 days through stem cell-driven proliferation to maintain barrier integrity (1–3). Disruption of this critical homeostatic mechanism, is a hallmark of inflammatory bowel disease (IBD) (4, 5), a chronic inflammatory condition encompassing Ulcerative Colitis and Crohn’s Disease (6, 7). During IBD, innate immune cells, particularly macrophages play a pivotal and dual role (8–10). They are central orchestrators of both the initiation of destructive inflammation and the resolution of damage, a functional plasticity reflected in their broad categorization into pro-inflammatory M1 and anti-inflammatory, tissue-reparative M2 polarization states (11, 12).

The balance between macrophage activation states is essential for maintaining immune homeostasis. Classical M1 polarization, triggered by stimuli such as IFN-γ and LPS, promotes the secretion of pro-inflammatory cytokines, including IL-6, thereby amplifying inflammation and aggravating tissue injury (13). In contrast, alternative M2 polarization, driven by IL-4 and related cues, is characterized by elevated production of anti-inflammatory mediators such as IL-10, which attenuate immune responses and facilitate tissue repair (11, 14). The JAK-STAT signaling cascade, particularly STAT3, serves as a central regulatory node in macrophage function; however, its effects are highly context-dependent, capable of mediating both pro- and anti-inflammatory outcomes (15, 16). This functional duality implies that upstream modulators defining the specificity and magnitude of JAK-STAT signaling remain to be elucidated.

AF6 (MLLT4) is a highly conserved scaffolding protein that functions as a master regulator of cell adhesion and cytoskeletal organization, playing a pivotal role in maintaining epithelial homeostasis (17–19). Its complete knockout leads to embryonic lethality, underscoring its fundamental importance in development (20). Although AF6 is classically characterized as a junctional protein, increasing evidence suggests that scaffold proteins can function beyond structural roles by organizing signaling complexes and facilitating signal transduction. Notably, AF6 has been shown to interact with multiple signaling molecules and participate in the regulation of intracellular signaling pathways beyond cell-cell junctions. In the context of immune cells, however, whether AF6 contributes to signaling cascades that govern macrophage activation remains largely unknown. Given the central role of macrophages in intestinal inflammation and the shared signaling pathways between epithelial and immune cells, we hypothesized that AF6 may regulate macrophage polarization and thereby influence intestinal inflammatory responses.

Here, we identify AF6 as a novel and critical immune regulator in macrophages. We found that AF6 expression was significantly upregulated in macrophages upon inflammatory challenge. Using a myeloid-specific AF6 knockout model, we demonstrate that loss of AF6 alleviates DSS-induced colitis by impaired M1 polarization under inflammatory conditions. Integrative single-cell RNA sequencing and mechanistic validation revealed that AF6 acts as a key scaffold facilitating the JAK2-STAT3 interaction, thereby licensing the pro-inflammatory M1 program. Disruption of AF6 disrupts this signaling hub leading to attenuated JAK/STAT3 pathway activation and enhanced epithelial repair. Collectively, our study defines a previously unknown paradigm for AF6 as a gatekeeper of macrophage plasticity and identifies the AF6-STAT3 axis as a promising therapeutic target for IBD.

## 2 Materials and methods

### 2.1 Animals

The LysM-Cre mice were a generous gift from Lu Wei (Shanghai Institute of Nutrition and Health). LysM^Cre^AF6^f/f^ (MKO) mice were generated by crossing the LysM^Cre^ mice with AF6^f/f^ mice for specific deletion of AF6 in myeloid on a C57BL/6J background. Littermate AF6^f/f^ mice lacking the Cre transgene were used as controls in all experiments. Genomic DNA was extracted from mouse tail snips and subjected to PCR using the following primers: LysM Wild type: TTACAGTCGGCCAGGCTGAC, LysM Mutant: CCCAGAAATGCCAGATTACG, LysM Common: CTTGGGCTGCCAGAATTTCTC. All mice were males, 8–12 weeks of age and randomized into experiment. All experimental mice were genotyped by PCR to confirm the presence of the floxed AF6 allele and the Cre transgene. Animals were maintained under a 12-h/12-h light/dark cycle at 25 °C and were provided with free access to water and food. For DSS-induced acute colitis model, The standard drinking water was replaced with drinking water containing 2.5%(w/v) DSS (obtained as Dextran sulfate sodium salt, colitis grade (36,000-50,000), Catalog No. 02160110-CF; MP Biomedicals, Santa Ana, CA, USA). Animals were maintained on DSS-water for 7 days, during which the drinking water was changed once daily. The body weight, stool consistency and gross bleeding were monitored daily during the development of acute colitis. The disease activity index (DAI) scores were calculated as described previously (21). Briefly, no weight loss was registered as 0, weight loss of 1-5% from baseline was assigned 1 point, 6-10% 2 points, 11-20% 3 points, and more than 20% 4 points. For stool consistency, 0 points were assigned for well-formed pellets, 2 points for pasty and semiformed stools that did not adhere to the anus, and 4 points for liquid stools that did adhere to the anus. For bleeding, 0 was assigned for no blood, 2 points for bleeding, and 4 points for gross bleeding.

### 2.2 Western blot

Total proteins were extracted from colon tissues and cells using Radioimmunprecipitation Assay (RIPA) buffer with protease inhibitors, and the concentration was detected by BCA protein assay kit according to the manufacturer’s protocol (Thermofisher, Shanghai, China). Equal amounts of protein (20-30 μg) were separated via New Semet Express-cast PAGE color gel (8% SDS-PAGE) electrophoresis and electrophoresed onto PVDF membranes by wet transfer at 100 V for 150 min at 4 °C. The membranes were blocked with 5% skim milk for 1 h, and incubated with primary antibodies incubated at 4 °C overnight. After washed with 1×TBST solution for 3 times, the PVDF membrane was incubated with the corresponding secondary antibody for 2 h. Then the PVDF membrane was washed with 1×TBST solution 3 times again, and the exposure was analyzed and sorted out. The primary antibodies were as follows: anti-AF6 (BD Biosciences; Cat#610732), anti-heat shock protein 90 (HSP90) (Cell Signaling Technology; Cat #4875S), anti-p-p65 (Cell Signaling Technology; Cat #3033), Occludin (Abmart; Cat #TD7504), anti- E-cadherin (BD Biosciences; Cat#610182), anti-STAT3 (Cell Signaling Technology; Cat #9139), anti-p-STAT3(Y705) (Cell Signaling Technology; Cat #9145). HSP90 or GAPDH was used as a loading control.

### 2.3 Co-immunoprecipitation

Co-IP assays were performed using bone marrow-derived macrophages (BMDMs). After Total proteins of cells were extracted with cell lysis buffer, with protease and phosphatase inhibitors. the protein concentration was determined by the BCA method and 1mg protein was taken for subsequent immunoprecipitation. Each sample was incubated with 1 µg antibody overnight at 4 °C, then incubation with 30 μl of Protein G Beads at 4 °C for 2 h. Normal IgG was used as a negative control to assess nonspecific binding, and input samples (5-10% of total lysate) were included to verify protein expression levels. The immunocomplexes were washed 5 times with IP lysis buffer, then resuspended with 2×SDS Sample Buffer for Western blotting. Immunoprecipitated proteins were then analyzed by Western blotting using the indicated antibodies.

### 2.4 RNA isolation and real-time PCR

Total RNA was isolated from the cells and mice colon tissues using Trizol reagent (Takara, Cat#9108) and was reverse-transcribed into cDNA using the PrimeScript™RT reagent kit (Takara, Cat#R037A). cDNA was used for RT-PCR amplification using the Hieff UNICON^®^ Power qPCR SYBR Green Master Mix (Yeasen #11196ES03). The primers used to amplify each target gene were shown in **Table 1**.

**Table 1:** Summary of primer sequences.

Gene	Sequence
Actin	Forward: CTACCTCATGAAGATCCTGACC
	Reverse: CACAGCTTCTCTTTGATGTCAC
AF6	Forward: ACTCCCTCTATGAAGTGCATGT
	Reverse: TAAGGACGAATCGACCCTCTC
IL-6	Forward: CAACGATGATGCACTTGCAGA
	Reverse: CTCCAGGTAGCTATGGTACTCCAGA
IL-1b	Forward: CCAAAAGATGAAGGGCTGCT
	Reverse: ACAGAGGATGGGCTCTTCTT
TNF-a	Forward: ACTCCAGGCGGTGCCTATGT
	Reverse: GTGAGGGTCTGGGCCATAGAA
INOS	Forward: GGAGTGACGGCAAACATGACT
	Reverse: TCGATGCACAACTGGGTGAAC
CCL2	Forward: AGCTGTAGTTTTTGTCACCAAGC
	Reverse: GTGCTGAAGACCTTAGGGCA
Gob5	Forward: GGCTCATCATTGCCTAGAGG
	Reverse: CCCAGAATTACCAAGTGAGTCCT
Muc2	Forward: TGTGGAACCGGGAAGATG
	Reverse: GACCACAGGTATGGTTCTGGA
Occludin	Forward: TTGAAAGTCCACCTCCTTACAGA
	Reverse: CCGGATAAAAAGAGTACGCTGG
ZO-1	Forward: GATGAGCGGGCTACCTTA
	Reverse: TGGAGACTGCGTGGAATG
Claudin1	Forward: TGCCCCAGTGGAAGATTTACT
	Reverse: CTTTGCGAAACGCAGGACAT
IL-10	Forward: GCTCTTACTGACTGGCATGAG
	Reverse: CGCAGCTCTAGGAGCATGTG

### 2.5 Isolation and culture of colon organoids

Colons from WT mice were dissected under sterile conditions, flushed with ice-cold PBS containing 2% ampicillin/streptomycin (Gibco), and inverted to expose the crypts. Tissues were incubated in Cell Recovery Solution (Corning, Cat#354253) for 40–50 min with continuous pipetting to release crypts, then filtered through a 70-μm strainer. After centrifugation (300×g, 5 min, 4 °C), the pellet was washed 4–5 times with organoid cleaning medium (DMEM/F12 with 1% penicillin/streptomycin, 1% GlutaMAX, and 1% HEPES) at 250×g (2 min, 4 °C) until the supernatant was clear. Crypts were resuspended in Matrigel (Corning, Cat #354230) at 200 crypts/50 μL and plated in 24-well plates. After Matrigel solidification (10 min at 37 °C), wells were overlaid with 500 μL organoid complete medium: 1:1 DMEM/F12 and L-Wnt3A conditioned medium (Fuxiang Biotech) supplemented with 20% FBS, 1% penicillin/streptomycin, 500 ng/mL R-Spondin (R&D Systems, Cat #3474-RS), 50 ng/mL EGF (Sino Biological, #50482-M01H), 100 ng/mL Noggin (Peprotech, Cat #250-38-20), and 10 μM Y-27632 (Tocris, Cat #1254). For IL-10 stimulation, organoid culture medium containing 10 ng/mL IL-10 (Peprotech, Cat #310-10) and cultured for 3 days. For co-culture with supernate of BMDM from AF6^fl/fl^ and MKO mice, during the BMDM culture process described, on day 7 we harvested the supernatant from the BMDMs, filtered it, and mixed it with fresh culture medium in a 1:1 ratio. We then added this conditioned medium on day 3 of the organoid culture, photographed the samples at indicated time points for observation, and measured their diameters.

### 2.6 Isolation of lamina propria immune cells

The mice were sacrificed and the colons were collected. They were cut into 1 cm segments, and sequentially incubated with PBS supplemented with 1 mM dithiothreitol (DTT) for 10 min, 30 mM ethylenediaminetetraacetic Acid (EDTA) for 10 min at 37 °C to remove epithelial cells. Subsequently, the residuary parts were incubated with RPMI 1640 medium supplemented with 200 U/mL Collagenase 8 and 150 μg/mL DNase I for 90 min at 37 °C, filtered by using nylon mesh cell strainer. The pellet was re-suspended in 4 mL 40% Percoll and 2.5ml 80% Percoll was added into bottom slowly, and centrifuged at 1000g for 20 min. Finally, collect the middle cells and stained for flow cytometry.

### 2.7 BMDMs isolation and cell culture

BMDMs were isolated and cultured as we previously reported. Briefly, BMDMs were collected from six-to eight-week-old mice. Red Blood Cell Lysis Buffer (Yeasen, Cat#40401ES76) was used to remove erythrocytes. The remaining cells were incubated in RPMI 1640 (Gibco) containing 1% penicillin/streptomycin (Gibco), 10% fetal bovine serum (FBS) (Gibco), and 10 ng/ml recombinant mouse M-CSF (Novoprotein, Cat#0331488) for 7 days to promote differentiate into bone marrow-derived macrophages. After 7 days, the differentiated macrophages were cocultured with interleukin 4 (IL-4;10 ng/ml) (Ppeprotech, Cat#214-14) or lipopolysaccharide (LPS; 100 ng/ml) at the indicated time points. For M1 polarization, BMDMs or RAW 264.7 cells were treated with LPS (100 ng/ml) for 24 hours. For STAT3 inhibitor assay, RAW 264.7 cells were treated with the STAT3 inhibitor Stattic (10 µM) together with LPS for 24 hours.

### 2.8 Flow cytometry

For cell-surface antigen staining, the cells isolated from spleens, colonic lamina propria or harvested from *in vitro* culture were incubated with the LIVE/DEAD^®^ Fixable Aqua Dead Cell Stain Kit (Catalog No. L34966; Thermo Fisher Scientific; stain dilution, 1:1000) for 30 min at 4 °C in the dark, then incubated in Fc block Anti-Mouse CD16/CD32 (Catalog No. 16-0161-86; Invitrogen/Thermo Fisher Scientific) 1:200 for 15 min at 4 °C in the dark and fluorescently labeled antibodies for 1 h at 4 °C in the dark as follows: CD19-PE (Invitrogen; Cat#12-0193-81), CD3-FITC(Invitrogen; Cat#11-0031-85), CD4- eFluor450 (Invitrogen; Cat#11-0041-82), CD4- BV421 (Invitrogen; Cat#48-0041-82)CD8- PE (Invitrogen; Cat#12-0081-82), FOXP3 PE(Biolegend, Cat#320007), CD45-APC (Invitrogen; Cat#17-0451-82), F4/80-FITC (Biolegend; Cat#123107), F4/80- eFluor 450 (Invitrogen; Cat#48-4801-82) F4/80-PE (Biolegend; Cat#123110), CD206-PE (Invitrogen; Cat#12-2061-82), CD86-PE (eBioscience; Cat#12-0862-82) CD11b-FITC (Invitrogen; Cat#11-0112-85), IL-10- PE(Biolegend; Cat#12-7101-81), Ly6C-APC (Biolegend; Cat#128035), Ly6G -PE (ebioscience; Cat#12-5931-82), NK1.1- PE (biolegend; Cat#108707), MHCII -PE-Cyanine7 (Biolegend; Cat#107630), CD11c -APC (Invitrogen; Cat#17-0114-82).

For intracellular staining, cells were first surface stained, then permeabilized using the Cytofix/Cytoperm kit (BD PharMingen #554722) according to the manufacturer’s instructions. The permeabilized cells then were subjected to intracellular staining by 30 minutes of co-incubation with antibodies against the indicated intracellular factors. For stimulation of intracellular factors, cells were resuspended in cell stimulation medium (consisting of RPMI 1640 medium, 10% FBS, 1% penicillin/streptomycin, β-mercaptoethanol, 1% HEPES, 1% acetone sulfate, 1% GlutMAX, 0.1% phorbol 12-myristate 13-acetate (PMA), 0.1% ionomycin, and 0.1% brefeldin A (BFA)) and distributed to the individual wells of round-bottom 96-well plates. The plates then were incubated in a cell culture incubator for 4–6 hours before staining as above.

### 2.9 RNA-seq and data analysis

For RNA-seq, total RNA collected from isolated colon tissues of DSS-induced AF6^f/f^ and MKO mice was extracted by Trizol. The quality of RNA samples and cDNA libraries were constructed and sequenced by Majorbio Biotech. GESA and cluster analysis were performed by Majorbio Biotech (Shanghai,China).

### 2.10 Single-cell RNA sequencing

Lamina propria immune cells were collected from isolated colon tissues of DSS-induced AF6^f/f^ and MKO mice. Single-cell suspensions were submitted to Shanghai Majorbio Bio-Pharm Biotechnology Co., Ltd. (Shanghai, China) for library construction, sequencing, and bioinformatic analysis. Single-cell RNA sequencing libraries were generated using the Chromium Single Cell 3’ Library & Gel Bead Kit v3.1 (10x Genomics, Pleasanton, CA), following the manufacturer’s instructions. All functions were run with default parameters, unless specified otherwise.

For QC, the filtering criteria for this run using Seurat are as follows: Mitochondria (filtered), ribosomes, and red blood cells (animals only). Statistical analysis of the data is performed using CellRanger, with reference to the Mouse Gene Database. The number of captured cells per sample ranged from 9,996 to 10,375, with a total of 20,371 cells captured. The median number of genes per cell ranged from 2,032 to 2,058, and the range of valid barcodes was between 96.1% and 96.4%. First, PCA dimensionality reduction was performed using the Seurat package. Subsequently, data mining was conducted using the SingleR database to identify cell clusters, analyze cluster-specific genes, and perform gene set analysis. The RNA rate analysis was performed using version 0.2.4 of the scVelo software, and the subclusterTree analysis was performed using version 0.4.4 of the clustree software with the default parameters as specified on the website.

### 2.11 TIMER2.0 analysis

For TIMER2.0 analysis, Gene expression data and immune infiltration scores were obtained from the TIMER2.0 database (https://compbio.cn/timer2/). The “immune association” module was used to assess the correlation between MLLT4 expression and infiltration levels of M1 macrophages. Spearman’s rank correlation coefficient was calculated, and p < 0.05 was considered statistically significant. All analyses were performed using default parameters as recommended by the TIMER2.0 documentation.

### 2.12 Analysis of public scRNA-seq datasets

Using R, we analyzed mouse inflammatory bowel disease (IBD) data from the Gene Expression Omnibus (GEO) database (GSE264408) and human single-cell RNA sequencing (scRNA-seq) data from databases GSE266616 and GSE236459 to investigate the expression of the AF6 gene across different cell populations. First, the data was loaded and processed using the Seurat package. Cell selection criteria were established according to literature standards to exclude low-quality cells, such as those with a mitochondrial gene proportion >25%, in order to remove potential dead or low-quality cells. Since scRNA-seq data may exhibit batch effects, Harmony was employed for batch correction to reduce technical bias and improve data comparability. After data normalization, cell clustering was performed, and dimension reduction visualization using t-SNE or UMAP was conducted to explore the distribution of different cell populations. Cell populations were classified based on the expression of macrophage-specific marker genes such as Adgre1 (F4/80), Cd68, and Cd163 to screen for and identify IBD-associated macrophage populations. Use FetchData to extract the expression profile of the AF6 gene across all cell types and observe expression differences among different cell clusters.

### 2.13 Statistics and reproducibility

Unless stated otherwise, data are presented as means ± SEM. The data were analyzed using GraphPad Prism (San Diego, CA, version 8.0). Pairwise comparisons between groups were conducted using two-tailed non-paired Student’s t-tests. p values < 0.05 were considered statistically significant. For histology scores and DAI, Mann-Whitney U test was used. In figures, differences are indicated as *p < 0.05, **p < 0.01, ***p < 0.001, or not significant (ns; p ≥ 0.05).

### 2.14 Ethical statement

All animal use strictly adhered to federal regulations and guidelines set forth by the Institutional Animal Care and Use Committee of the Shanghai Institute of Nutrition and Health, Chinese Academy of Sciences (SINH-2024-ZLX-2). This study was not subject to medical research involving human subject. Therefore, it did not require written informed consent from patients.

## 3 Results

### 3.1 AF6 expression was increased in inflammatory macrophages

To investigate AF6 expression and its role in macrophages, we first measured AF6 mRNA levels in various immune cell subsets. Myeloid cells, especially macrophages, showed high AF6 expression, while T cells exhibited moderate levels (**Supplementary Figure S1A**). Publicly available single-cell RNA sequencing dataset of colonic tissues from healthy and colitis mice (GSE264408) revealed increased AF6 expression in macrophages during colitis (**Supplementary Figure S1B**). Consistently, public scRNA-seq dataset of colonic macrophages from Crohn’s disease patients (GSE266616 and GSE236459) exhibited higher AF6 levels in inflamed versus non-inflamed regions (**Supplementary Figure S1C**). To validate these findings, we treated BMDMs and the macrophage cell line RAW 264.7 cells with LPS. Western blot analysis confirmed significant upregulation of AF6 after LPS stimulation, supporting its potential involvement in inflammation-associated macrophage activation (**Supplementary Figure S1D**).

### 3.2 AF6 deficiency in myeloid cells has no effect on colon homeostasis under steady-state conditions

To investigate the function of AF6 in macrophages, we generated myeloid-specific AF6 knockout mice (LysM^Cre^AF6^f/f^) as described in Methods, in which AF6 deletion is most efficient in macrophages and confirmed by Western blot and qRT-PCR (**Supplementary Figures S2A, B**). Importantly, baseline AF6 expression was substantially lower in neutrophils compared to macrophages (**Supplementary Figure S1A**), suggesting that the functional consequences of LysM-Cre-driven AF6 deletion are likely dominated by effects in macrophages. Throughout this study, we focused our analyses on macrophage-enriched compartments, and the term “MKO” refers to these mice. As shown in **Supplementary Figure S2C**, there was no evident difference in colonic histological analysis between the MKO mice and its control (**Supplementary Figure S2C**). In addition, we found no difference in colon length between both groups of mice (**Supplementary Figure S2D**). Moreover, AF6 KO did not affect the levels of fluorescein isothiocyanate (FITC)-dextran in serum (**Supplementary Figure S2E**), suggesting AF6-specific deletion in macrophages did not influence colonic homeostasis under general condition.

We then assessed whether AF6 deletion affected immune cell development. MKO mice displayed no significant differences in the frequency of CD4^+^ T cells, CD8^+^ T cells, B cells, DCs, and macrophages in the spleen (**Supplementary Figure S2F**) or colon (**Supplementary Figure S2G**) compared to AF6^f/f^ controls.

### 3.3 AF6 deficiency in myeloid cells attenuates DSS-induced colitis

**Figure 1:**
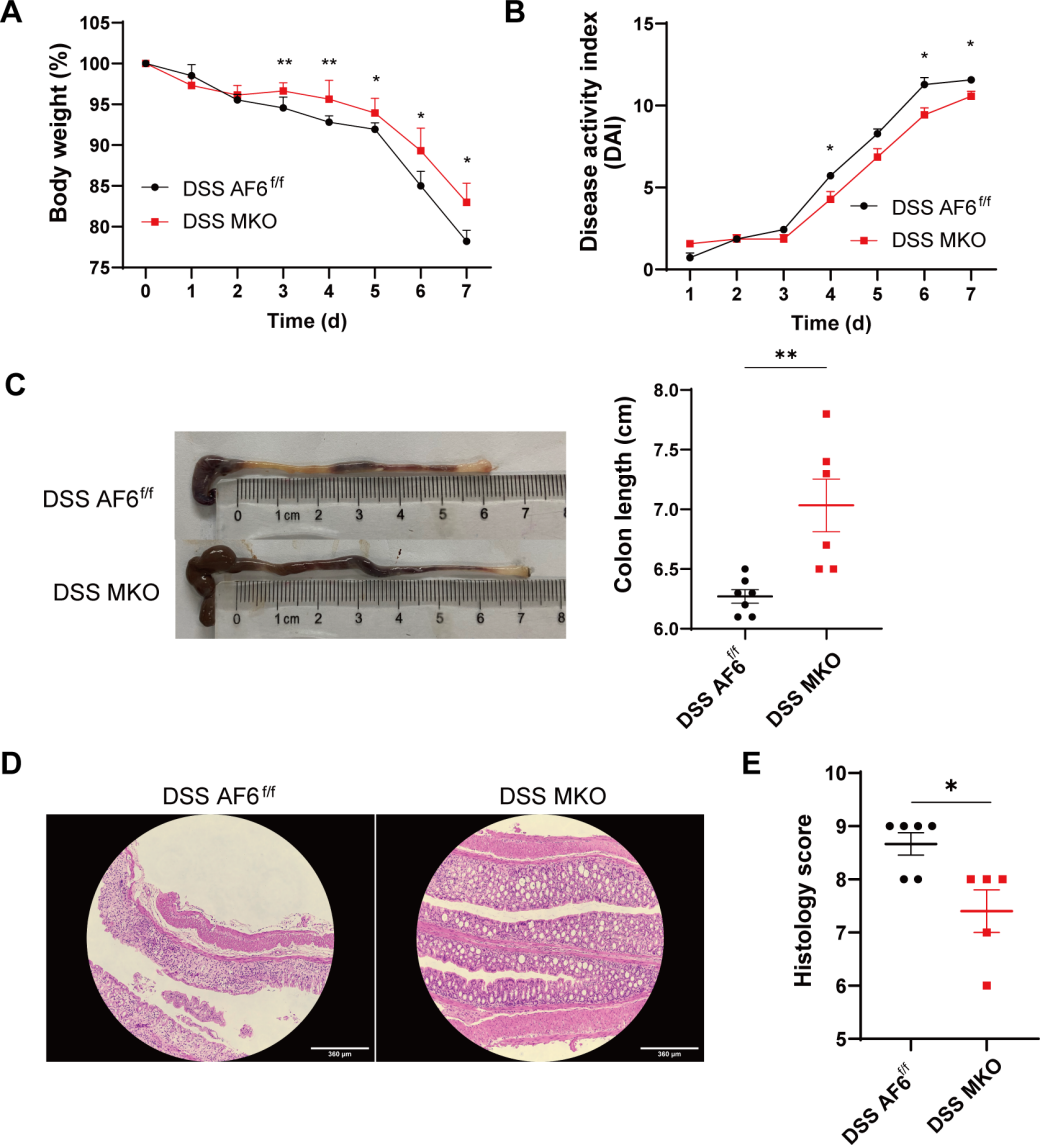
AF6 deficiency in macrophages attenuates DSS-induced colitis. AF6^f/f^ and MKO mice were administered water or 2.5% DSS for 7 days to induce acute colitis. **(A, B)** Body weight changes and clinical score were assessed daily in the DSS-induced colitis model as described in the Methods section (n = 6). **(C)** Gross morphology of colons from AF6^f/f^ and MKO mice. Colon lengths were measured on day 7 (n=7). **(D)** H&E-stained images of colon sections. Scale bars: 360 μm (whole colon section). **(E)** Histological score assessing colitis severity in AF6^f/f^ and MKO mice on day 7 post-DSS administration. Data are expressed as mean ± SEM. Pairwise comparisons between groups were conducted using two-tailed non-paired Student’s t tests. *p < 0.05, **p < 0.01.

To determine whether AF6 KO in macrophages affected colonic inflammatory progression, we applied DSS-induced colitis model. MKO and AF6^f/f^ mice were treated with 2.5% DSS for 7 consecutive days to injury gut mucosal barrier and induce inflammation, and thus to induce colitis. MKO mice exhibited significantly less body weight loss, diarrhea, and rectal bleeding compared to their control (**Figures 1A, B**). MKO mice also showed longer colon lengths after DSS treatment (**Figure 1C**). Histological analysis revealed decreased inflammatory cell infiltration and preserved mucosal architecture in MKO mice, as shown by H&E staining (**Figure 1D**). Consistently, MKO mice displayed markedly lower histological colitis scores than DSS-treated AF6^f/f^ mice (**Figure 1E**).

### 3.4 AF6 deficiency in myeloid cells led to reduced intestinal inflammation

**Figure 2:**
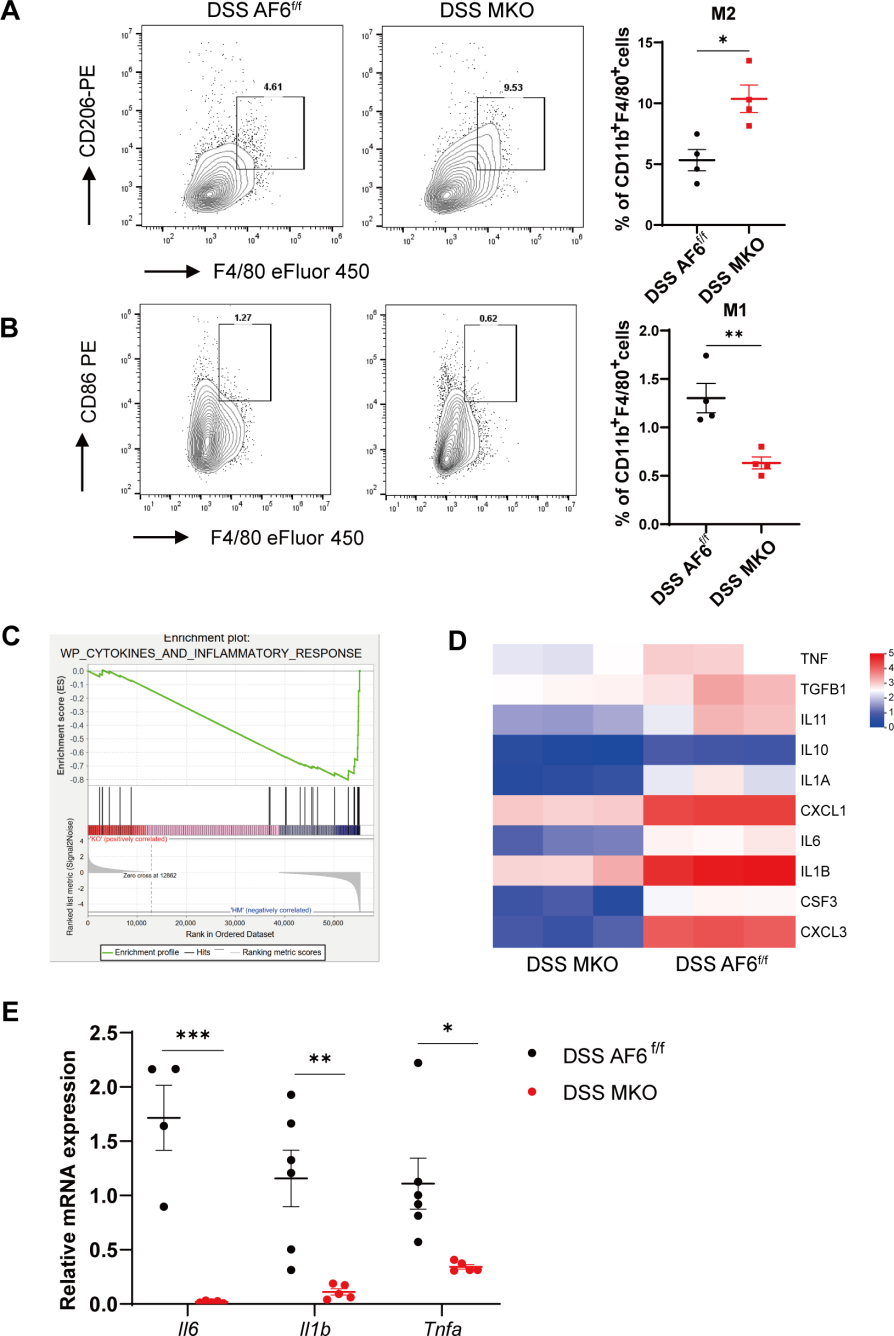
Macrophages-specific deletion of AF6 reduce DSS-induced intestinal inflammation. **(A, B)** Representative flow cytometric plots for M2 (CD206^+^, **(A)** and M1 (CD86^+^, **(B)** macrophages gated on CD45^+^CD11b^+^F4/80^+^ living cells isolated from CLP from AF6^f/f^ and MKO mice on days 7 after DSS challenge. **(C)** Gene Set Enrichment Analysis (GSEA) analysis of differentially expressed genes in AF6^f/f^ and MKO mice on days 7 after DSS challenge (n=3). **(D)** Heatmap showing the expression levels of inflammatory cytokines and chemokines. **(E)** The qRT-PCR data of key pro-inflammatory cytokines. Data are expressed as mean ± SEM. Pairwise comparisons between groups were conducted using two-tailed non-paired Student’s t tests. *p < 0.05, **p < 0.01, ***p < 0.001.

To evaluate the impact of AF6 deletion on immune cell infiltration during colitis, immune cells in lamina propria (LP) were isolated and analyzed by flow cytometry. Notably, MKO mice had a significantly higher percentage of M2 macrophages in the colon lamina propria compared to AF6^f/f^ mice (**Figures 2A, B**). Accordingly, the percentage of M1 macrophages was decreased significantly (**Figure 2B**). These changes in macrophage subsets occurred without significant alterations in other immune cell populations, including the *MLLT4 (AF6) expression* CD4^+^ T cells, Treg cells, and neutrophils (**Supplementary Figure S3**).

To explore global gene expression changes, RNA sequencing (RNA-seq) was performed on colonic tissues from DSS-treated AF6^f/f^ and MKO mice (**Supplementary Figure S4A**). The analysis revealed downregulation of inflammatory gene expression, including IL-6, IL-1α and TNF-α, while IL-10, an immunoregulatory cytokine with anti-inflammatory properties was increased in MKO mice (**Figures 2C, D**, **Supplementary Figure S4B**). It is worth noting that the IL-6-JAK-STAT3 pathway was less enriched in MKO mice compared with AF6^f/f^ mice. (**Supplementary Figures S4C, D**). In particular, the expression of key inflammatory and chemotactic cytokines, including IL-1β, IL-6, and TNF-α, was significantly reduced in AF6-deficient mice following DSS exposure (**Figure 2E**). These findings indicated that AF6 deficiency in macrophages led to reduced intestinal inflammation, probably by regulating macrophage polarization.

### 3.5 AF6 deficiency decreases M1 macrophage polarization *in vitro*

**Figure 3:**
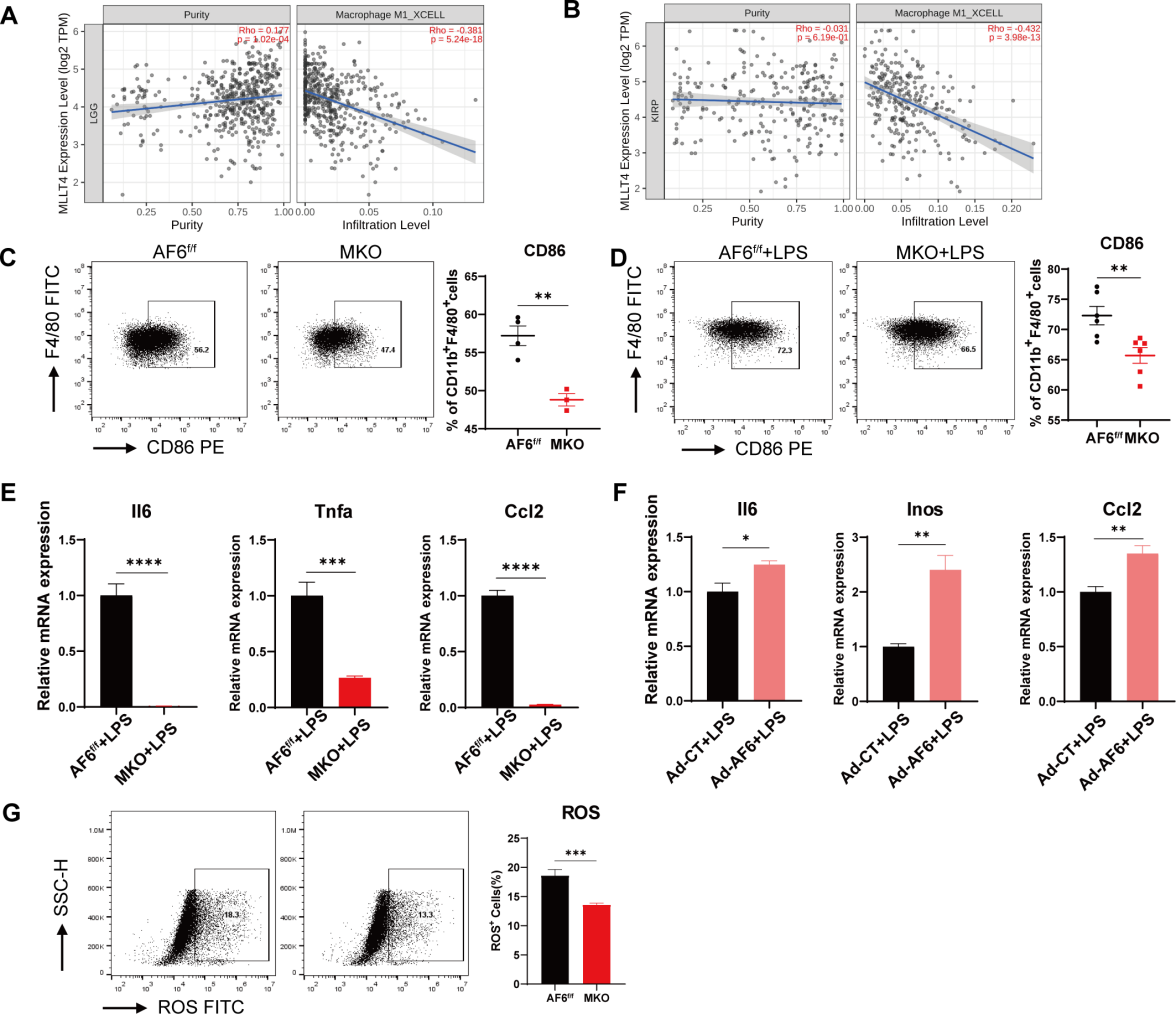
AF6 deficiency decreases macrophage M1 polarization *in vitro*. **(A, B)** Scatter plots showing the relationship between the expression of AF6 and the infiltration level of M1 macrophages in the TCGA LGG (n=516) and KIRP (n=290) cohort. The infiltration levels were obtained from the TIMER2.0 database. **(C, D)** Flow cytometry analysis of CD86 in BMDMs from WT and MKO mice under non-treated (NT) conditions and 24h after LPS stimulation. **(E)** The mRNA expression of M1 maker genes was detected in LPS-treated BMDM from MKO and its control mice. **(F)** The mRNA expression of various inflammatory genes in LPS-treated BMDMs infecting with adenovirus overexpressing AF6 (Ad-AF6) or its control (Ad-CT). **(G)** Flow cytometry analysis of ROS expression in BMDMs isolated from MKO and its control AF6^f/f^ mice. Data are expressed as mean ± SEM. Pairwise comparisons between groups were conducted using two-tailed non-paired Student’s t tests. *p < 0.05, **p < 0.01, ***p < 0.001, ****p < 0.0001.

To assess whether AF6 directly regulates macrophage polarization, correlation analysis using TIMER2.0 datasets revealed that MLLT4 (AF6) expression was negatively correlated with M1 macrophage infiltration (**Figures 3A, B**). Flow cytometry of BMDMs revealed that MKO cells exhibited fewer CD86^+^ (M1) macrophages under non-treated (NT) conditions (**Figure 3C**). Upon LPS stimulation, AF6-KO BMDMs still showed inhibitory effect on M1 polarization (**Figure 3D**). Consistently, AF6 deficiency gave rise to downregulated M1-related inflammatory gene expression (IL-6, IL-1β and TNF-α) in LPS-treated BMDMs (**Figure 3E**). Conversely, AF6 overexpression promoted these LPS-induced M1 inflammatory gene expression (**Figure 3F**). ROS is the representative marker for classic M1 macrophages. Of note, we observed that ROS production was significantly lower in MKO BMDMs (**Figure 3G**). Together, these findings indicated a clear role of AF6 in promoting macrophage M1 polarization, pointing to a potential role for AF6 blockade in limiting colitis and supporting tissue repair.

### 3.6 Single-cell sequencing reveals AF6 deletion regulates macrophage subpopulations and inhibits their IL-6/JAK/STAT3 pathway

**Figure 4:**
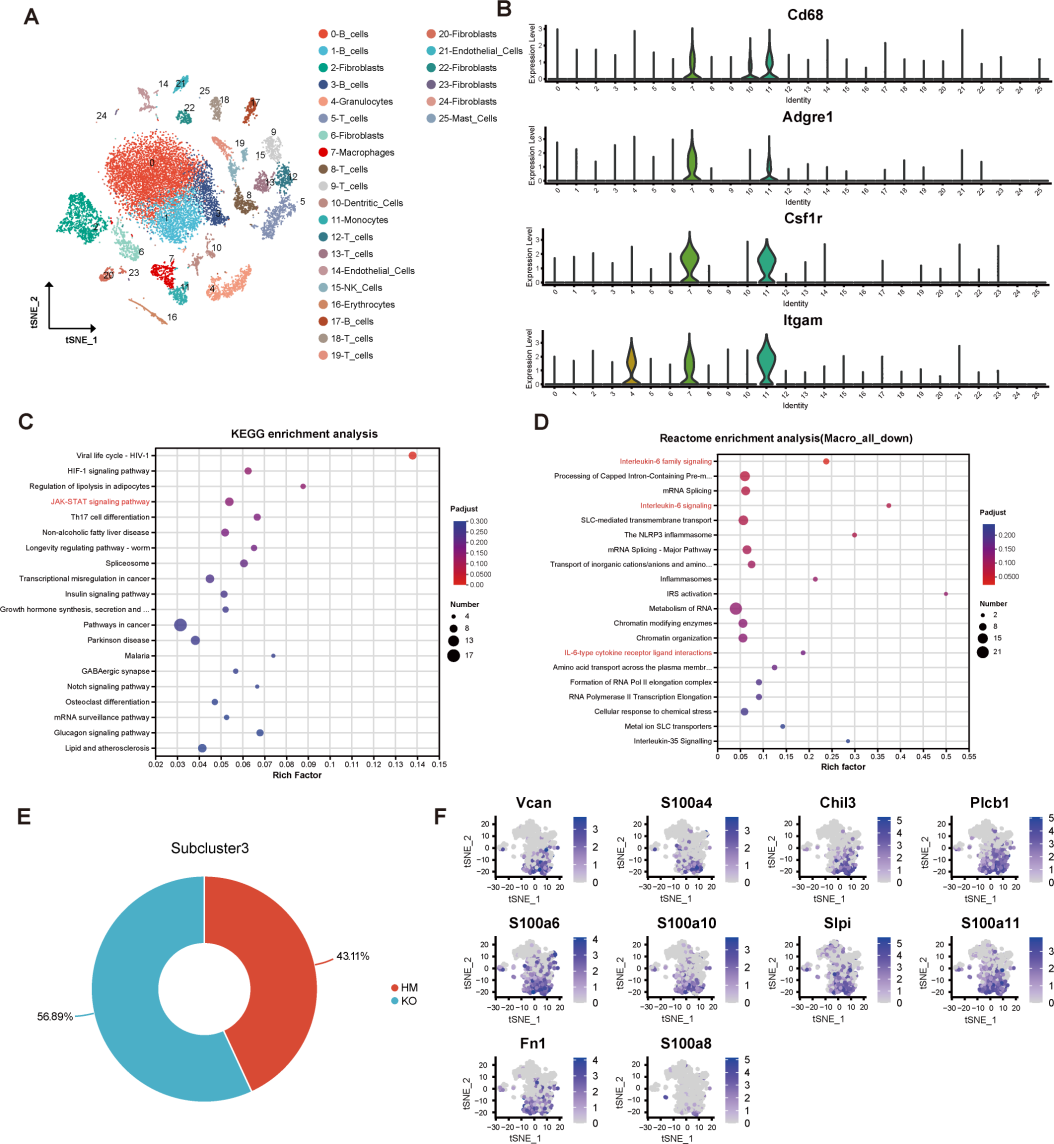
Single-cell sequencing reveals AF6 deletion regulates macrophage subpopulations and their IL-6/JAK/STAT3 pathway. **(A)** t-SNE plots showing a total of 26 cell clusters cell types from DSS-treated AF6^f/f^ and MKO mice. **(B)** Violin plots showing the expression levels of classical macrophage markers CD68, Adgre1, Csf1R and Itgam across various cell clusters. **(C, D)** KEGG **(C)** and Reactome **(D)** enrichment analysis of IL-6-JAK-STAT pathway in macrophages, highlighting its downregulation in the context of AF6 deficiency. **(E)** Donut chart illustrating the composition of macrophages subcluster3 between AF6 KO and AF6^f/f^ mice. **(F)** t-SNE plots display expression patterns of specific genes associated with macrophage polarization.

To further investigate the role of AF6 in macrophage function, we performed a new scRNA-seq on colonic lamina propria immune cells from DSS-treated AF6^f/f^ and MKO mice. Clustering analysis identified 26 distinct cell populations (**Figure 4A**). Based on automated classification using publicly available databases, we identified clusters 7 and 11 as macrophage populations (**Figure 4B**; **Supplementary Figure S5A**). KEGG and Reactome enrichment analysis of downregulated genes in MKO macrophages revealed significant suppression of the JAK-STAT signaling pathway (**Figures 4C, D**) and it was also confirmed by GSEA (**Supplementary Figures S5B**, **S6B**). Further subclustering identified seven macrophage subsets, among which subcluster 3 showed a trend of expansion in MKO mice (**Figure 4E**; **Supplementary Figure S5C**). The top markers of this subpopulation were associated with anti-inflammatory functions (**Figure 4F**). RNA Velocity showed that subcluster 3 was in the middle to late stages of differentiation (**Supplementary Figure S5D**). Here, we confirmed the systemic downregulation of inflammatory responses conferred by AF6 deletion. Given the critical role of the JAK-STAT3 pathway in regulating inflammatory responses in immune cells, we focused subsequent studies on elucidating its involvement in AF6-mediated macrophage regulation.

### 3.7 AF6 promotes JAK-STAT3 pathway activation via strengthening JAK-STAT3 binding

**Figure 5:**
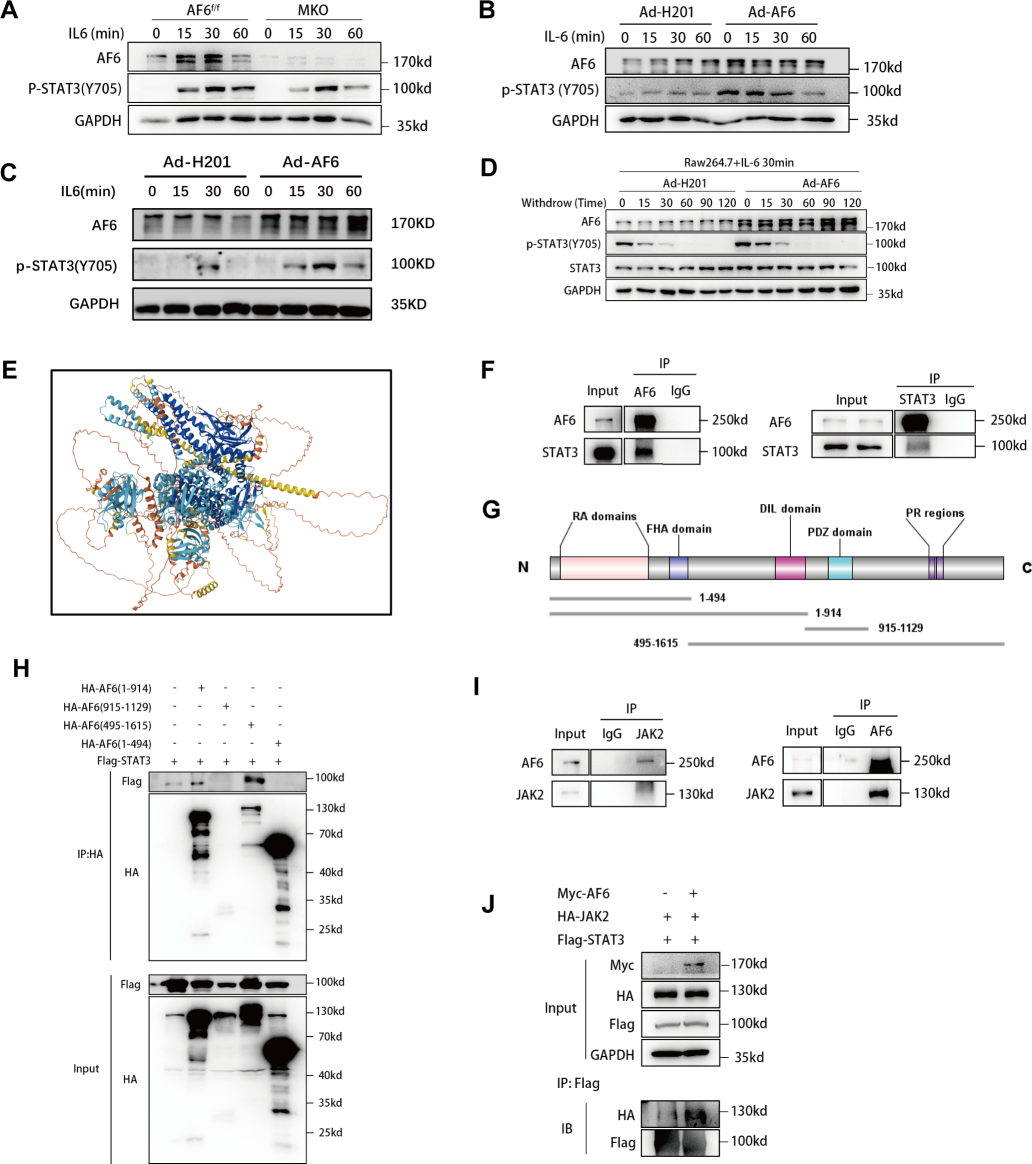
AF6 deficiency inhibits M1 polarization by downregulating the JAK2-STAT3 pathway. **(A)** BMDM from AF6^f/f^ and MKO mice were stimulated with IL-6 (10 ng/mL) for the indicated times (0, 15, 30, and 60 minutes). STAT3-Tyr705 phosphorylation were assessed by western blot. **(B, C)** BMDMs **(B)** or RAW 264.7 cells **(C)** expressing AF6 by adenovirus infect were stimulated with IL-6 (10 ng/mL) for the indicated times. STAT3-Tyr705 phosphorylation were assessed by western blot. **(D)** RAW264.7 cells expressing AF6 by adenovirus infect were treated with IL-6 for 30 min. Cell were then harvested at the indicated time to detect the dephosphorylation trand of STAT3. **(E)** Structure prediction of potential binding sites of AF6 and STAT3 by AlphaFold3. **(F)** Co-IP assay showed AF6 and STAT3 have endogenous mutual binding. **(G)** Schematic diagram of the structural domain of AF6 protein. **(H)** HA-AF6 fragment plasmid and Flag-STAT3 plasmid were co-transfected into 293T cells, and their interacting domains were detected by Co-IP assay. **(I)** Co-IP assay showed endogenous mutual binding of AF6 and JAK2. **(J)** HA-JAK2 plasmid was co-transfected into 293T cells, together with plasmid Flag-STAT3 and Myc-AF6, and their interaction was detected by Co-IP assay.

To elucidate the role of AF6 in macrophage activation, we stimulated BMDMs with IL-6. In AF6-deficient cells, Tyr705 phosphorylation was significantly diminished following short-term IL-6 exposure (**Figure 5A**). Conversely, AF6 overexpression via adenovirus potentiated the Tyr705 phosphorylation in BMDMs (**Figure 5B**) and RAW 264.7 cells (**Figure 5C**), further confirming its role in STAT3 activation. Cytokine withdrawal experiments showed comparable dephosphorylation kinetics between both groups, suggesting that AF6 promotes STAT3’s phosphorylation without affecting its dephosphorylation dynamics (**Figure 5D**). Together, these results suggest that AF6 facilitates JAK/STAT3 signaling by enhancing STAT3 Tyr705 phosphorylation, thereby promoting M1-like macrophage polarization.

We hypothesized that AF6 might directly interact with STAT3. To test this, we first employed AlphaFold3 structural predictions, which identified potential binding sites between AF6 and STAT3-related signaling proteins (**Figure 5E**). Consistent with this prediction, co-immunoprecipitation (Co-IP) assays confirmed endogenous AF6-STAT3 binding in mouse BMDMs (**Figure 5F**). To further map the interaction domain, we generated truncated AF6 mutants and co-expressed them with STAT3 in HEK293T cells. Co-IP analysis demonstrated that AF6 primarily interacts with STAT3 via its C-terminal domains (**Figures 5G, H**). Since JAK2 is a canonical upstream kinase regulating STAT3 phosphorylation, we proposed that AF6 may enhance STAT3 phosphorylation by facilitating JAK2-STAT3 complex formation. Indeed, Co-IP assays confirmed endogenous AF6-JAK2 interaction in BMDMs (**Figure 5I**). Notably, pull-down assays revealed that Myc-AF6 overexpression significantly strengthened STAT3-JAK2 binding (**Figure 5J**), supporting our hypothesis.

### 3.8 JAK-STAT3 pathway activation contributes to AF6-associated M1 polarization and pro-inflammatory gene expression in macrophages

**Figure 6:**
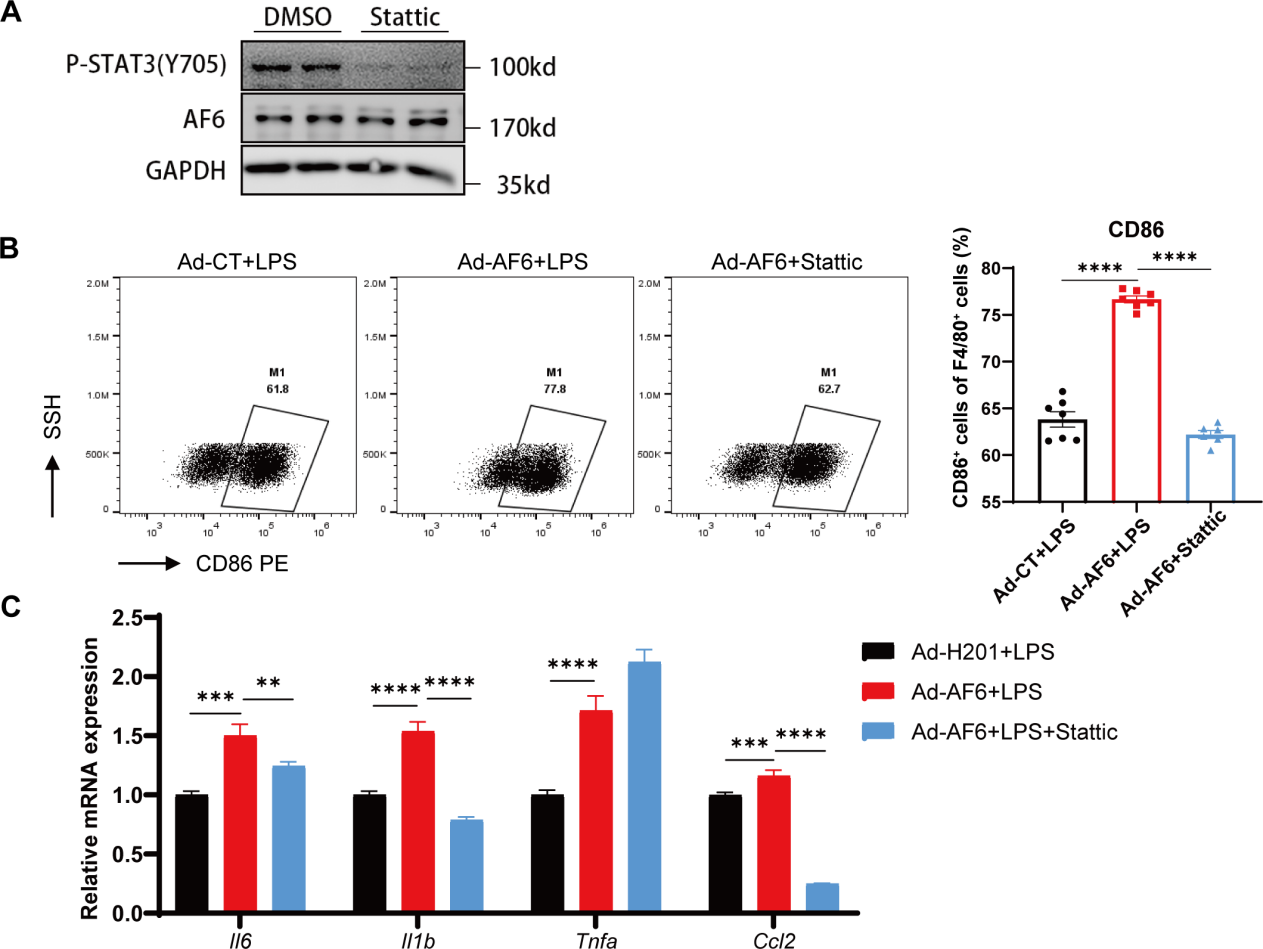
Pharmacological inhibition of STAT3 activation decreases AF6-induced M1 polarization. **(A)** Western blot detection for STAT3 inhibitor Stattic upon STAT3-Tyr705 phosphorylation. **(B)** Flow cytometry assay of Stattic’s effect upon macrophage M1 polarization induced by AF6 expression. **(C)** qRT-PCR was performed to detect the effect of Stattic upon M1-related pro-inflammatory gene expression in macrophages expressing AF6. Data are expressed as mean ± SEM. Pairwise comparisons between groups were conducted using two-tailed non-paired Student’s t tests. p < 0.05, **p < 0.01, ***p < 0.001, ****p < 0.0001.

Given that AF6 strengthens JAK2-STAT3 binding and promotes STAT3 phosphorylation (**Figure 5**), we next asked whether JAK-STAT3 activation is functionally required for AF6-induced M1 polarization. To address this, we used Stattic, a specific STAT3 inhibitor preventing STAT3 phosphorylation (22). RAW 264.7 cells were treated with Stattic and simultaneously induced M1-like polarization. Notably, Stattic treatment had no effect on AF6 protein levels, confirming that its inhibition of STAT3 is direct and AF6-independent (**Figure 6A**).

To assess whether AF6-mediated M1 polarization depends on STAT3 activation, we overexpressed AF6 in RAW 264.7 cells and co-treated them with Stattic and LPS. Flow cytometry analysis showed that AF6 overexpression significantly enhanced M1 polarization upon LPS stimulation, but this effect was significantly attenuated by Stattic (**Figure 6B**). Consistent with this, qRT-PCR analysis revealed that AF6 overexpression upregulated pro-inflammatory gene expression (IL-6, IL-1β, TNF-α, CCL2) after LPS stimulation, whereas Stattic treatment suppressed their expression (**Figure 6C**). Together, these findings indicate that AF6 promotes M1 macrophage polarization and pro-inflammatory gene expression, at least in part, through JAK-STAT3 activation.

### 3.9 Myeloid AF6 deficiency contributed to improved intestinal epithelial recovery

**Figure 7:**
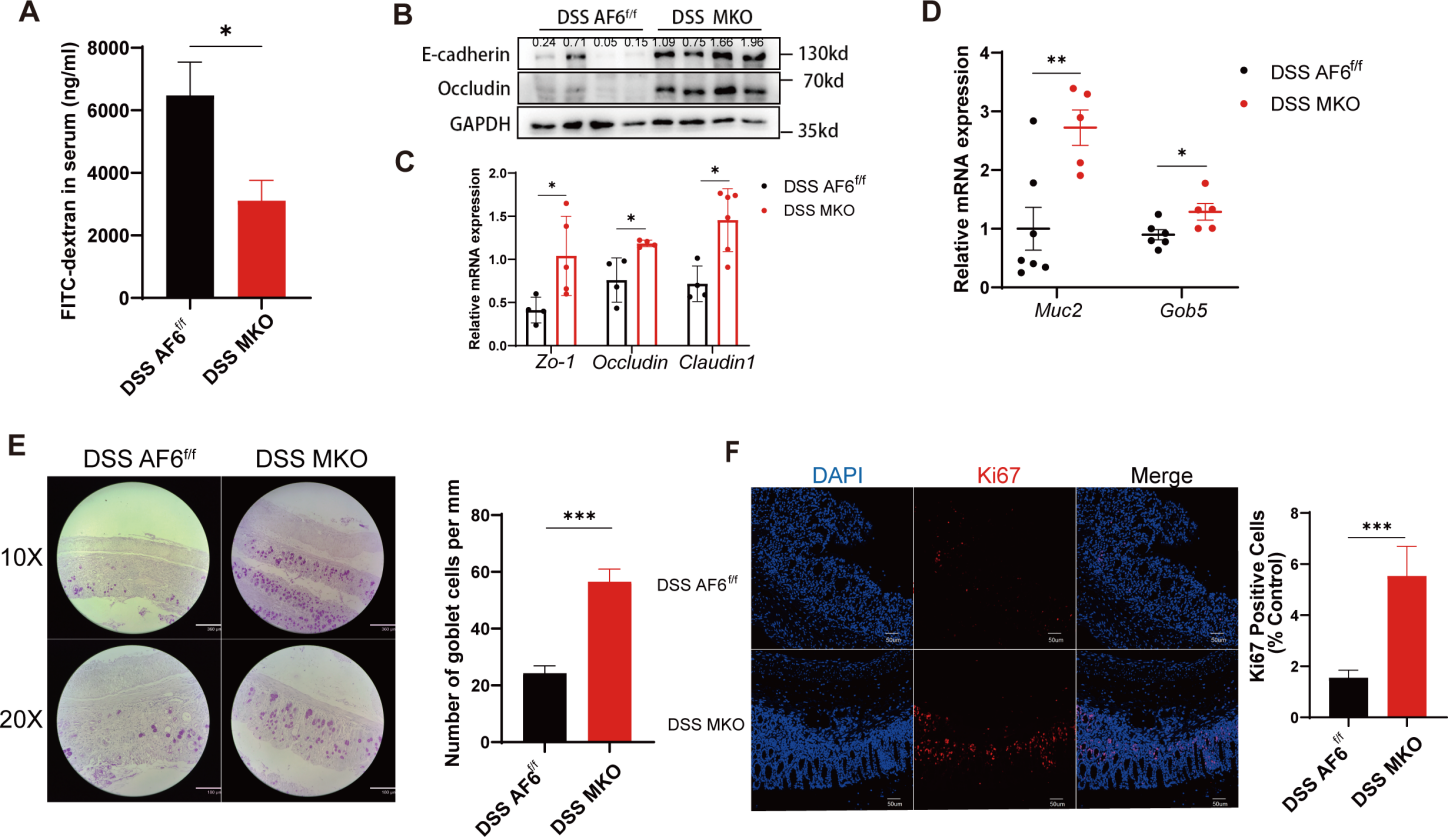
AF6 deficiency in macrophages promotes the recovery from DSS-induced colitis. **(A)** Intestinal permeability was assessed using FITC-dextran in mice 7 days after DSS challenge. **(B, C)** Western blot **(B)** and qRT-PCR analysis **(C)** of tight junction and epithelial adhesion genes in intestinal tissues of mice 7 days after DSS challenge. **(D)** Relative mRNA expression of goblet cell markers Muc2 and Gob5 were analyzed by qRT-PCR. **(E)** Acid-Schiff stain and quantify goblet cells in the intestine. **(F)** Quantification of Ki67^+^ epithelial cells from AF6^fl/fl^ and MKO mice challenged with 2.5% DSS (n = 5 biological replicates, 3 random images per mouse). Representative images are shown, scale bars, 10 mm. **(G)** BMDM from AF6^f/f^ mice were cultured with supernate of BMDM from AF6^fl/fl^ and MKO mice. Microscopic observation was performed at the indicated times, and the representative examination results were selected. **(H)** Statistical analysis of organoid diameters. **(I)** The β-catenin expression was detected in organoid by western blot. Data are expressed as mean ± SEM. Pairwise comparisons between groups were conducted using two-tailed non-paired Student’s t tests. p < 0.05, **p < 0.01, ***p < 0.001.

To evaluate whether myeloid-specific AF6 deletion affects barrier integrity, we measured intestinal permeability using a FITC-dextran-based assay. Serum FITC-dextran levels were significantly lower in MKO mice than in the WT control, indicating reduced intestinal permeability (**Figure 7A**). Western blot analysis of colonic tissues showed increased Occludin and E-cadherin expression in MKO mice (**Figure 7B**). This was further supported by elevated mRNA levels of Zo-1, Occludin, and Claudin1 in the knockout animals (**Figure 7C**), indicating improved intestinal barrier function in MKO mice rather than in their control. MKO mice also exhibited improved tissue repair, with increased goblet cell numbers and elevated mucin production. Expression of Muc2 and Gob5, key markers of mucus secretion, was markedly higher in MKO mice (**Figure 7D**), as confirmed by periodic acid-Schiff (PAS) staining, which showed increased and well-organized goblet cells (**Figure 7E**). Furthermore, Ki67 immunostaining revealed enhanced epithelial cell proliferation in MKO mice (**Figure 7F**), suggesting improved regenerative capacity. To further explore the mechanism, we employed an *in vitro* organoid-macrophage co-culture system. As expected, organoids co-cultured with AF6-deficient macrophages displayed significantly increased size and expansion capacity (**Supplementary Figure S6A**; **Figure 7B**). Western blot analysis revealed elevated β-catenin expression in organoids co-cultured with AF6-depleted macrophages (**Supplementary Figure 6C**), indicating enhanced Wnt signaling, a pathway known to be crucial for epithelial proliferation and regeneration. These findings suggest that in addition to alleviated inflammation, AF6 deletion in macrophages also contributed to better tissue repair, such as enhanced epithelial proliferation and stem cell activity.

### 3.10 Myeloid AF6 deficiency drives IL-10-dependent epithelial regeneration

The signaling pathway of IL-10/IL-10R plays a key role in regulating the intestinal macrophage phenotype in mice and humans (23–25). Studies have shown that IL-10 expression is rapidly up-regulated after intestinal mucosal damage in mice, which is critical for promoting mucosal repair and accelerating wound healing (26). In particular, IL-10 secreted by macrophages plays an important role in the damage repair process. Based on this background, we further explored the changes in IL-10 expression in macrophages. Given that AF6 deficiency promotes an anti-inflammatory macrophage phenotype, we investigated its effect on IL-10 production. QRT-PCR analysis revealed significantly elevated IL-10 expression in AF6-deficient BMDMs compared to control (**Supplementary Figure S7A**). Flow cytometry revealed an increased proportion of IL-10^+^ macrophages in MKO mice after 7 days of DSS challenge (**Supplementary Figure S7B**). To determine the functional significance of elevated IL-10 production, we examined its effect on epithelial regeneration using an intestinal organoid culture system. Supplementation with recombinant IL-10 significantly enhanced organoid growth compared to untreated controls (**Supplementary Figure S7C**). Mechanistically, Western blot of IL-10-treated organoids showed increased expression of β-catenin and c-Myc (**Supplementary Figure S7D**), key components of the Wnt signaling pathway. These results establish that macrophage-derived IL-10 contributes to epithelial renewal through activation of Wnt/β-catenin signaling.

## 4 Discussion

Our study uncovers a previously unappreciated role for the scaffold protein AF6 as a critical regulator of macrophage polarization in the context of intestinal inflammation. While AF6’s functions in epithelial polarity and stromal signaling are established, our data position it as a central node in innate immune pathogenesis. Through integrated single-cell RNA sequencing and mechanistic studies, we found that AF6 expression is upregulated in inflammatory macrophages and closely associated with the pro-inflammatory M1 phenotype in DSS-induced colitis. Crucially, we delineate the molecular mechanism: AF6 serves as a physical scaffold that stabilizes the JAK2-STAT3 complex, facilitating STAT3 phosphorylation at Tyr705 and thereby licensing the transcriptional program that sustains M1 polarization. This discovery adds a significant new layer to the understanding of STAT3 regulation, moving beyond cytokine-mediated activation to include a critical scaffolding mechanism that potentiates its signaling.

Beyond defining a cell-intrinsic role for AF6 in macrophage polarization, our work elucidates its impact on tissue repair through intercellular crosstalk. Using a co-culture system, we provide direct evidence that AF6-deficient macrophages create a pro-regenerative niche, markedly enhancing the proliferation and organoid-forming capacity of intestinal stem cells. This aligns with emerging paradigms of immunometabolic reprogramming in the intestinal niche. A potential concern is whether the observed phenotypes in MKO mice arise from loss of AF6 in macrophages or indirectly from epithelial AF6 dysfunction, given AF6’s well-established role in epithelial junctions. Several lines of evidence argue against this possibility. First, *in vitro* experiments using BMDMs demonstrated that AF6-deficient macrophages have cell-intrinsic defects in M1 polarization and JAK-STAT3 activation. Second, the LysM-Cre driver is not active in intestinal epithelial cells, and AF6 expression in colonic epithelium was unchanged in MKO mice. Third, baseline epithelial barrier integrity was normal in MKO mice. Collectively, these findings support a model in which AF6 functions cell-autonomously in macrophages to regulate their inflammatory and reparative programs. While our data point to IL-10 and subsequent Wnt/β-catenin activation as a key mediator, the full spectrum of mechanisms remains an exciting avenue for future exploration. For instance, recent work published in Cell Metabolism revealed that macrophages can supply polyamines to epithelia via an mTORC1-dependent pathway to fuel proliferation (4). It is therefore plausible that AF6 deletion skews macrophages toward a more reparative phenotype, orchestrating broader metabolic reprogramming that may involve the secretion of additional trophic factors synergizing with the IL-10/Wnt axis to drive epithelial renewal. Delineating the precise metabolite and factor exchange in this macrophage-epithelial unit will be a critical next step.

Although our findings robustly establish the AF6-JAK2-STAT3 axis in acute colitis, we acknowledge the inherent limitations of the DSS model. Inflammatory Bowel Disease in humans is a chronic and relapsing condition with a more complex etiology. Therefore, a crucial future direction will be to validate the role of myeloid AF6 in chronic colitis models, such as the adoptive T cell transfer model or repeated DSS cycles, which better recapitulate the fibrotic and relapsing aspects of human IBD. Despite this limitation, the DSS model remains a widely accepted and well-established system for studying innate immune responses and epithelial-immune crosstalk during intestinal inflammation. We recognize that further validation using additional models and human samples would strengthen the translational significance of our conclusions. This represents an important direction for future investigation. While our data demonstrate that AF6 physically interacts with JAK2 and STAT3 and that STAT3 inhibition attenuates AF6-associated M1 polarization, we acknowledge that pharmacological inhibition alone does not fully establish the JAK-STAT3 pathway as the exclusive mediator. Other downstream effectors or parallel pathways may also contribute. Future studies using genetic STAT3 ablation or reconstitution experiments will be required to further define the causal hierarchy. Furthermore, the upstream signals that govern AF6 expression in macrophages upon inflammatory challenge, and whether AF6 interacts with other signaling hubs beyond JAK-STAT, represent important unanswered questions that will deepen our understanding of its pleiotropic. Several aspects of AF6 regulation warrant further investigation. First, while we have demonstrated that AF6 expression is upregulated in macrophages upon LPS stimulation, suggesting a connection to TLR4-mediated inflammatory signaling, the precise transcriptional or post-transcriptional mechanisms responsible for this induction remain unknown. Whether transcription factors such as NF-κB and AP-1, directly regulate AF6 promoter, or whether post-translational mechanisms contribute to AF6 protein stability, awaits future study. Second, our analysis focused primarily on macrophages; we have not systematically examined AF6 expression in other myeloid cell types such as dendritic cells or neutrophils under inflammatory conditions. Given that LysM-Cre targets the entire myeloid lineage, it is possible that AF6 deficiency in these other populations may also contribute to the phenotypes observed in our MKO mice. Future studies using cell-type-specific knockout models (e.g., Cx3cr1-Cre for macrophages only) would help dissect the relative contributions of different myeloid subsets. Additionally, analysis of AF6 expression in human IBD samples across different immune cell compartments would strengthen the translational relevance of our findings.

In summary, through a combination of high-resolution transcriptomics and functional genetics, this study redefines AF6 from an epithelial polarity protein to a master immune regulator. We provide a mechanistic framework in which AF6, by scaffolding the JAK2-STAT3 complex, acts as a molecular rheostat controlling the balance between inflammatory and reparative macrophage states. This not only provides a novel theoretical foundation for IBD pathogenesis but also positions the AF6-STAT3 axis as a compelling and druggable target for therapeutic intervention, offering a promising strategy to simultaneously break the cycle of inflammation and promote mucosal healing in inflammatory diseases.

## Data Availability

The datasets analyzed GSE264408, GSE266616 and GSE236459 for this study can be found in the Gene Expression Omnibus (GEO) datasets.

## Supplementary material

Supplementary Figure 1 AF6 expression is increased in inflammatory macrophages. **(A)** Expression of AF6 mRNA in various immune cells, which includes bone-marrow-derived macrophages (BMDMs), peritoneal macrophages (PEMs), dendritic cells (MHCII^+^CD11c^+^), neutrophils (CD11b^+^Ly6G^+^Ly6C^low^), T cells (CD3^+^), and B cells (B220^+^). The expression levels of AF6 were normalized to the internal control β-actin. **(B)** UMAP findings illustrate the expression of AF6 in relation to single-cell clusters in macrophages, among mice with colitis and healthy controls. n= 6 per group. **(C)** UMAP findings illustrate the expression of AF6 in relation to single-cell clusters in macrophages, among patients with CD inflamed (CD), and non-inflamed (CD Health)controls. n= 6 per group. **(D)** AF6 protein expression was detected by western blot in BMDMs (upper panel) or RAW 264.7 cells (lower panel) treated with LPS or IL-4 for 24h.

Supplementary Figure 2 AF6 deficiency has no effects on macrophage development and colon tissues. **(A)** AF6 expression was detected by western blot in BMDMs (top) and PEMs (bottom) from MKO and AF6^f/f^ mice. **(B)** qRT-PCR analysis of AF6 mRNA expression in BMDMs from MKO and the control AF6^f/f^ mice. **(C)** Gross morphology of colons from MKO and AF6^f/f^ mice. **(D)** H&E-stained images of colon sections. Scale bar: 360 μm. **(E)** Intestinal permeability measured by fluorescein isothiocyanate (FITC)-dextran permeability. Representative flow cytometry data for immune populations in spleen **(F)** and colon. **(G)** derived from AF6^f/f^ and MKO mice. Data are expressed as mean ± SEM. Pairwise comparisons between groups were conducted using two-tailed non-paired Student’s t tests. ns, not significant.

Supplementary Figure 3 Flow cytometric analysis shows no significant differences in other immune cell populations. Representative flow cytometric plots isolated from CLP from AF6^f/f^ and MKO mice on days 7 after DSS challenge. ns, not significant.

Supplementary Figure 4 RNA-seq analysis shows attenuated inflammatory responses and reduced IL-6-JAK-STAT3 signaling in MKO mice. **(A)** Volcano plot showing the differential gene expression of AF6^f/f^ and MKO mice on day 7 post-DSS administration. (B-C) Gene set enrichment analysis (GSEA) demonstrated that both **(B, C)** pathways were significantly suppressed in AF6-deficient macrophages compared to controls. **(D)** Heatmap of representative differentially expressed genes associated with inflammation and cytokine signaling in DSS-treated macrophages from AF6^f/f^ and MKO mice.

Supplementary Figure 5 Single-cell RNA-seq analysis identifies macrophage clusters and reveals suppressed IL-6-JAK-STAT3 signaling in MKO mice. **(A)** Dot plot showing the expression profiles of representative cytokines and signaling-related genes across major immune cell types, including mast cells, NK cells, monocytes, dendritic cells, macrophages, T cells, granulocytes, and B cells. Dot size represents the percentage of cells expressing the gene, and color intensity indicates the average expression level. **(B)** Gene set enrichment analysis (GSEA) indicating significant suppression of the HALLMARK_IL6_JAK_STAT3_SIGNALING pathway in KO macrophages compared to controls. **(C)** Bar plot illustrating the proportions of macrophage subpopulations between AF6-deficient (KO) and control (HM) groups, showing an altered distribution of immune populations **(D)** RNA velocity analysis reveals the differentiation trajectories of macrophage subpopulations.

Supplementary Figure 6 Work model of the role of macrophages AF6 in colitis. AF6 as a critical regulator of macrophage polarization in the context of intestinal inflammation.
