# Supplementary figures and images for: AF6 orchestrates macrophage polarization via JAK2-STAT3 signaling and supports intestinal regeneration by stimulating stem cell proliferation
Source: Front Immunol. 2026 Jun 19;17:1798313. doi: 10.3389/fimmu.2026.1798313 (PMC13327902; doi:10.3389/fimmu.2026.1798313)

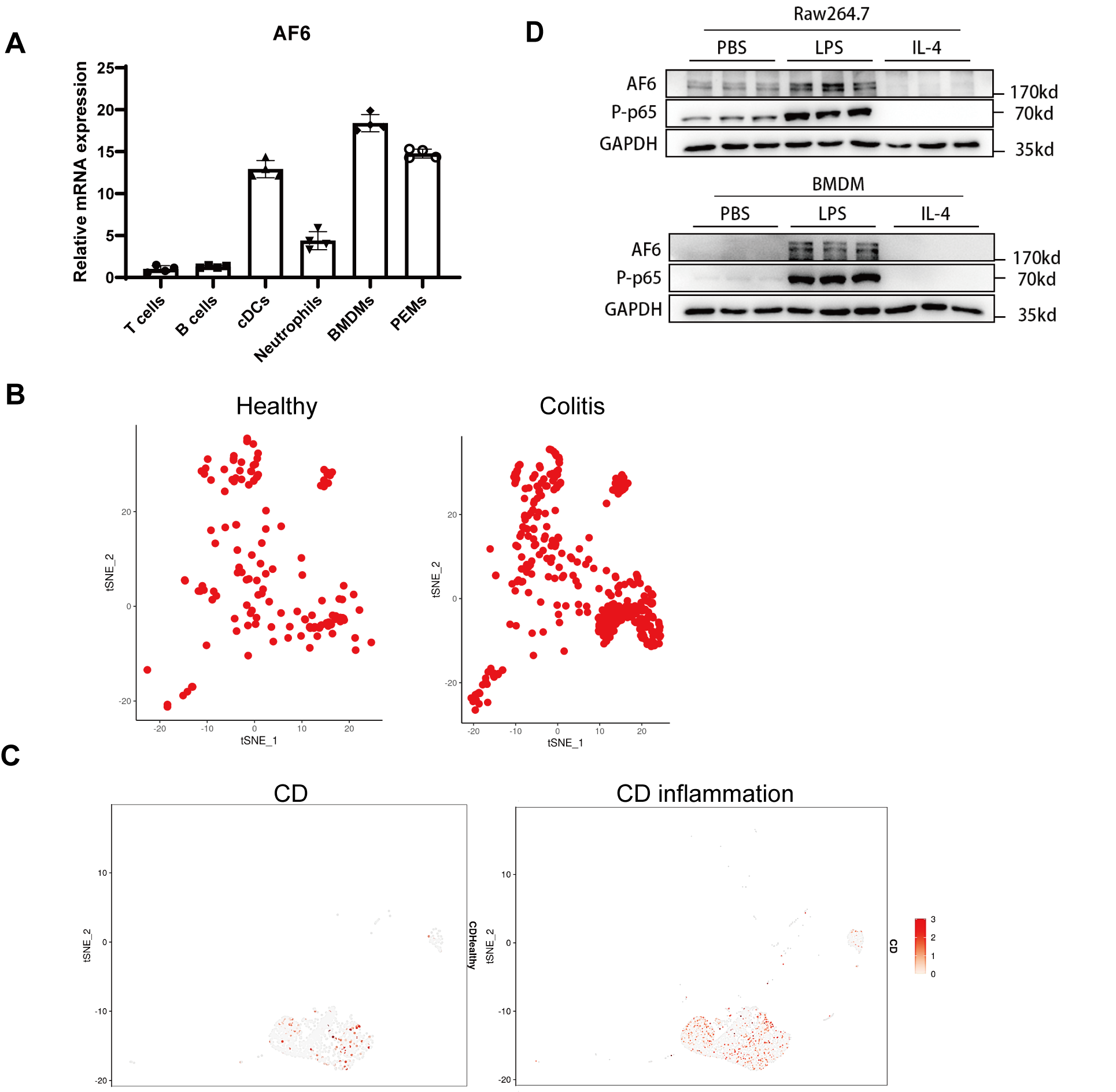

Supplement: Supplementary Figure 1 — AF6 expression is increased in inflammatory macrophages. (A) Expression of AF6 mRNA in various immune cells, which includes bone-marrow-derived macrophages (BMDMs), peritoneal macrophages (PEMs), dendritic cells (MHCII+CD11c+), neutrophils (CD11b+Ly6G+Ly6Clow), T cells (CD3+), and B cells (B220+). The expression levels of AF6 were normalized to the internal control β-actin. (B) UMAP findings illustrate the expression of AF6 in relation to single-cell clusters in macrophages, among mice with colitis and healthy controls. n= 6 per group. (C) UMAP findings illustrate the expression of AF6 in relation to single-cell clusters in macrophages, among patients with CD inflamed (CD), and non-inflamed (CD Health)controls. n= 6 per group. (D) AF6 protein expression was detected by western blot in BMDMs (upper panel) or RAW 264.7 cells (lower panel) treated with LPS or IL-4 for 24h. [file Supplementaryfile1.zip › supp/figure s1.tif]

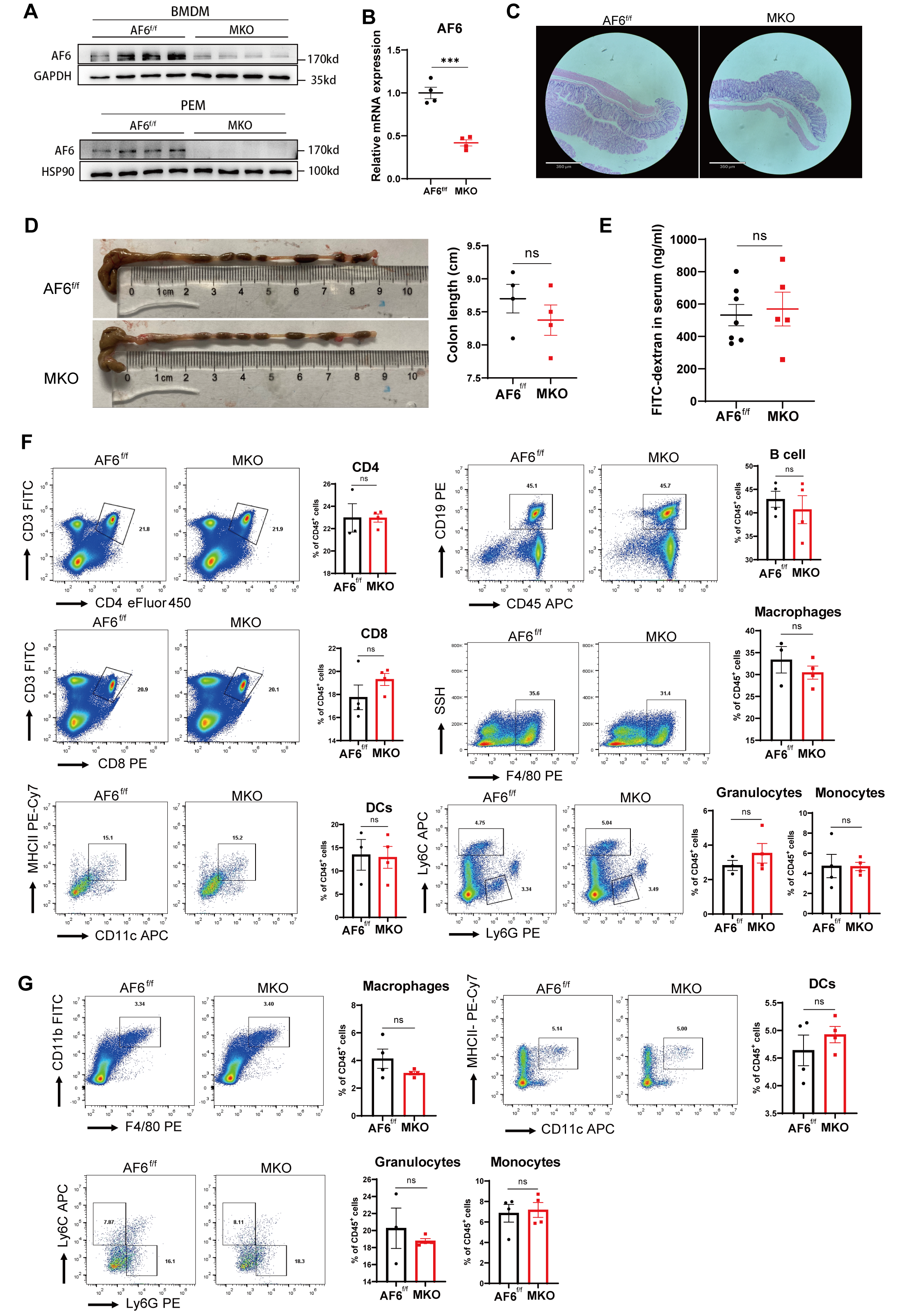

Supplement: Supplementary Figure 1 — AF6 expression is increased in inflammatory macrophages. (A) Expression of AF6 mRNA in various immune cells, which includes bone-marrow-derived macrophages (BMDMs), peritoneal macrophages (PEMs), dendritic cells (MHCII+CD11c+), neutrophils (CD11b+Ly6G+Ly6Clow), T cells (CD3+), and B cells (B220+). The expression levels of AF6 were normalized to the internal control β-actin. (B) UMAP findings illustrate the expression of AF6 in relation to single-cell clusters in macrophages, among mice with colitis and healthy controls. n= 6 per group. (C) UMAP findings illustrate the expression of AF6 in relation to single-cell clusters in macrophages, among patients with CD inflamed (CD), and non-inflamed (CD Health)controls. n= 6 per group. (D) AF6 protein expression was detected by western blot in BMDMs (upper panel) or RAW 264.7 cells (lower panel) treated with LPS or IL-4 for 24h. [file Supplementaryfile1.zip › supp/figure s2.tif]

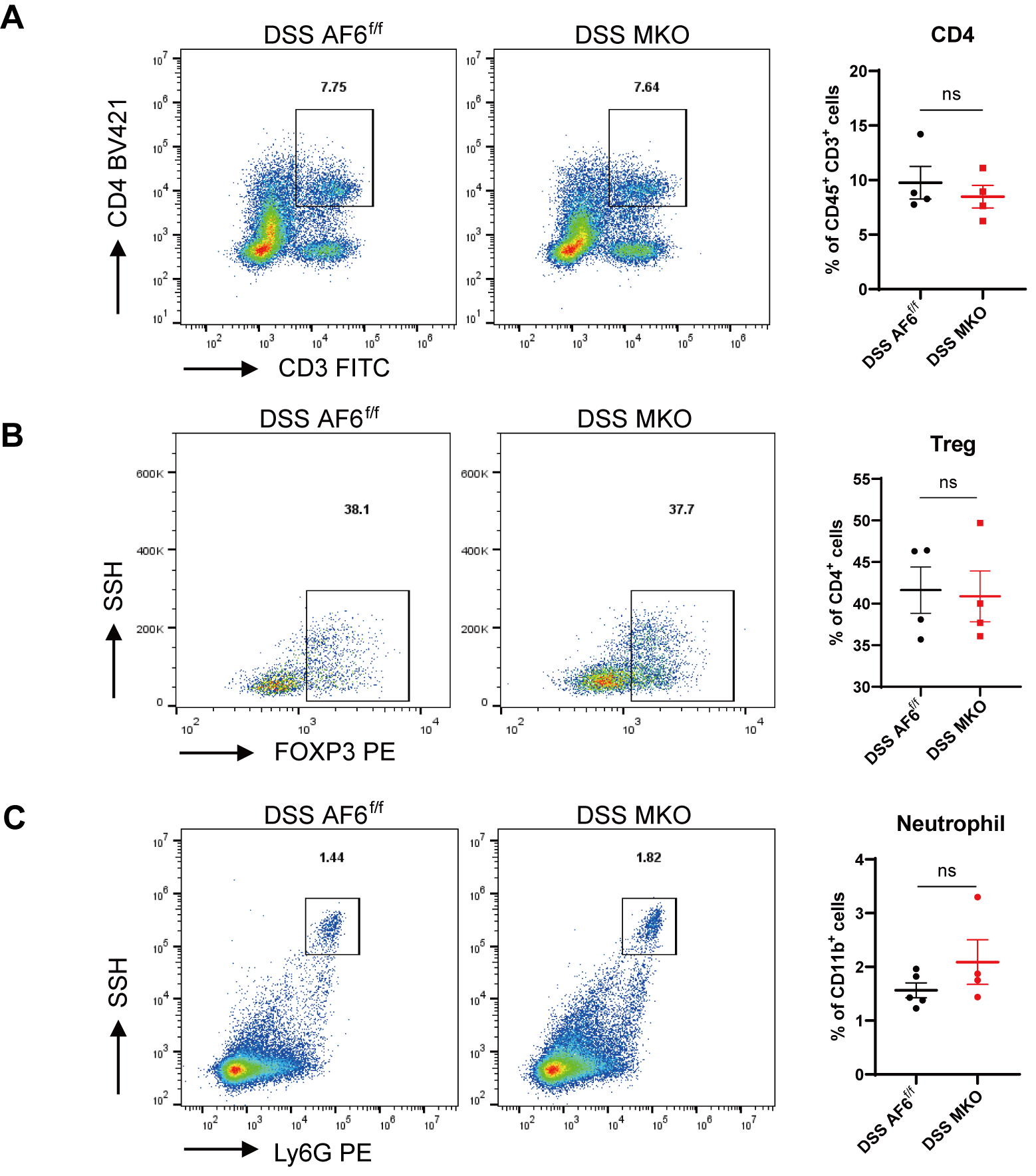

Supplement: Supplementary Figure 1 — AF6 expression is increased in inflammatory macrophages. (A) Expression of AF6 mRNA in various immune cells, which includes bone-marrow-derived macrophages (BMDMs), peritoneal macrophages (PEMs), dendritic cells (MHCII+CD11c+), neutrophils (CD11b+Ly6G+Ly6Clow), T cells (CD3+), and B cells (B220+). The expression levels of AF6 were normalized to the internal control β-actin. (B) UMAP findings illustrate the expression of AF6 in relation to single-cell clusters in macrophages, among mice with colitis and healthy controls. n= 6 per group. (C) UMAP findings illustrate the expression of AF6 in relation to single-cell clusters in macrophages, among patients with CD inflamed (CD), and non-inflamed (CD Health)controls. n= 6 per group. (D) AF6 protein expression was detected by western blot in BMDMs (upper panel) or RAW 264.7 cells (lower panel) treated with LPS or IL-4 for 24h. [file Supplementaryfile1.zip › supp/figure s3.tif]

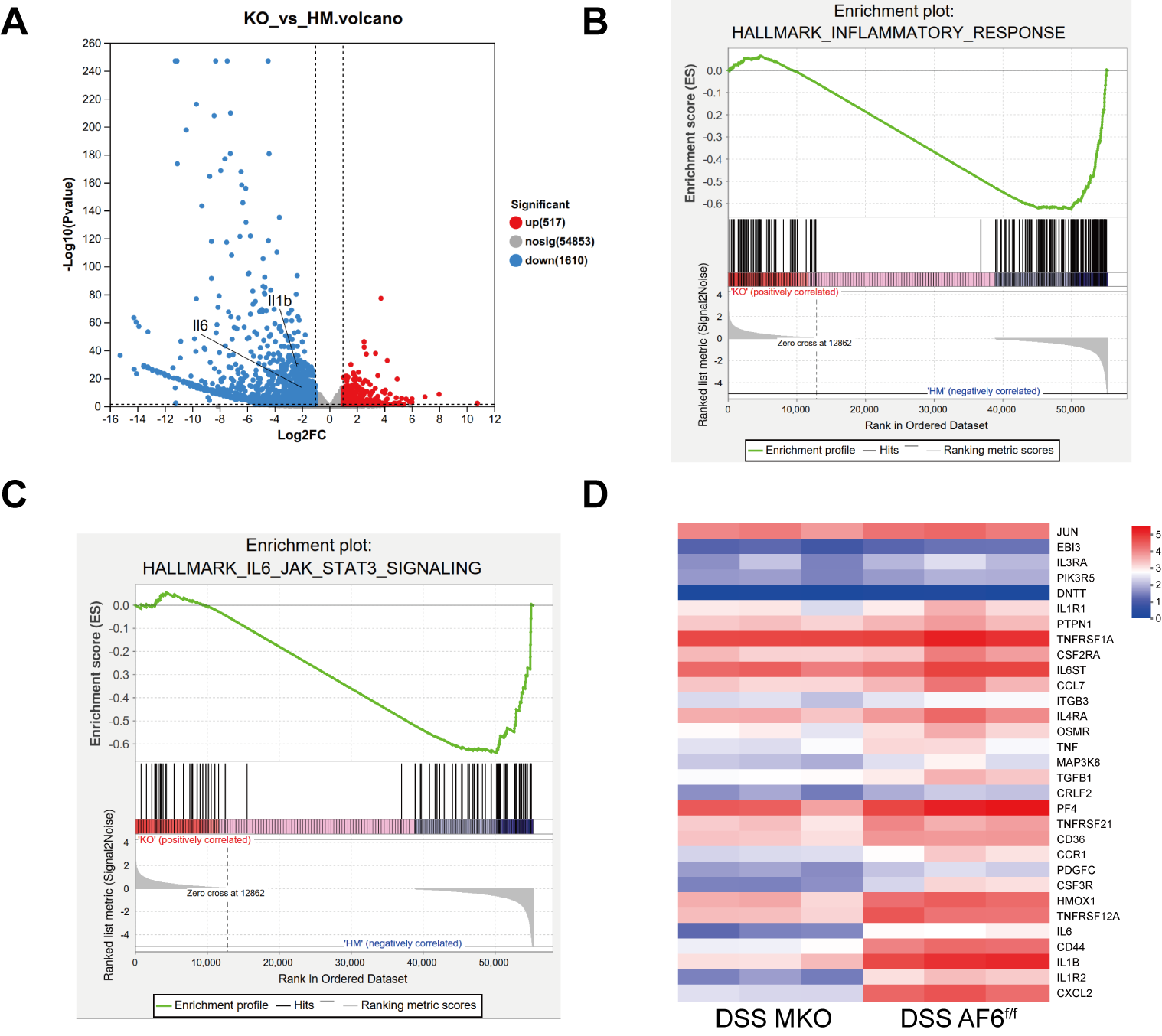

Supplement: Supplementary Figure 1 — AF6 expression is increased in inflammatory macrophages. (A) Expression of AF6 mRNA in various immune cells, which includes bone-marrow-derived macrophages (BMDMs), peritoneal macrophages (PEMs), dendritic cells (MHCII+CD11c+), neutrophils (CD11b+Ly6G+Ly6Clow), T cells (CD3+), and B cells (B220+). The expression levels of AF6 were normalized to the internal control β-actin. (B) UMAP findings illustrate the expression of AF6 in relation to single-cell clusters in macrophages, among mice with colitis and healthy controls. n= 6 per group. (C) UMAP findings illustrate the expression of AF6 in relation to single-cell clusters in macrophages, among patients with CD inflamed (CD), and non-inflamed (CD Health)controls. n= 6 per group. (D) AF6 protein expression was detected by western blot in BMDMs (upper panel) or RAW 264.7 cells (lower panel) treated with LPS or IL-4 for 24h. [file Supplementaryfile1.zip › supp/figure s4.tif]

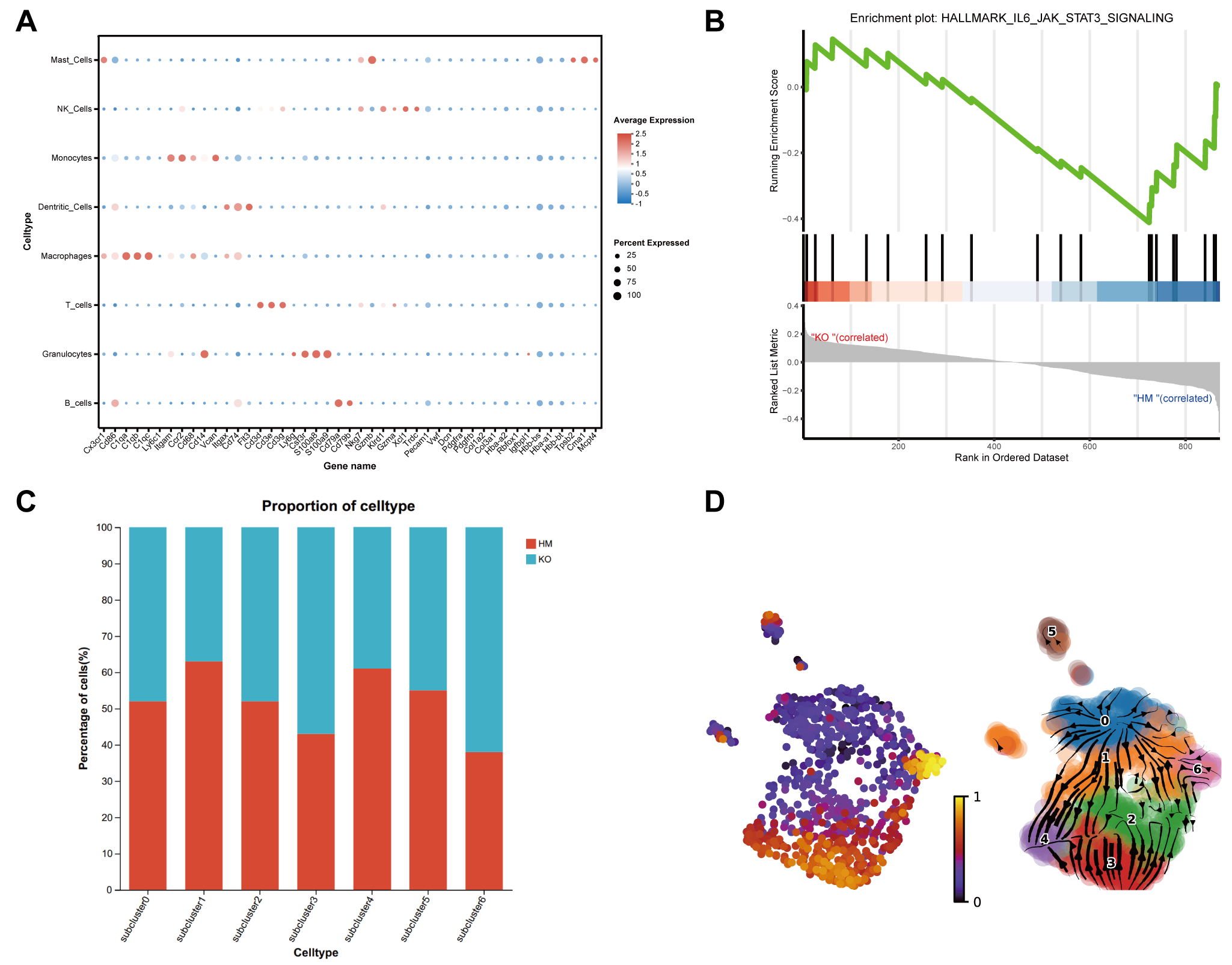

Supplement: Supplementary Figure 1 — AF6 expression is increased in inflammatory macrophages. (A) Expression of AF6 mRNA in various immune cells, which includes bone-marrow-derived macrophages (BMDMs), peritoneal macrophages (PEMs), dendritic cells (MHCII+CD11c+), neutrophils (CD11b+Ly6G+Ly6Clow), T cells (CD3+), and B cells (B220+). The expression levels of AF6 were normalized to the internal control β-actin. (B) UMAP findings illustrate the expression of AF6 in relation to single-cell clusters in macrophages, among mice with colitis and healthy controls. n= 6 per group. (C) UMAP findings illustrate the expression of AF6 in relation to single-cell clusters in macrophages, among patients with CD inflamed (CD), and non-inflamed (CD Health)controls. n= 6 per group. (D) AF6 protein expression was detected by western blot in BMDMs (upper panel) or RAW 264.7 cells (lower panel) treated with LPS or IL-4 for 24h. [file Supplementaryfile1.zip › supp/figure s5.tif]

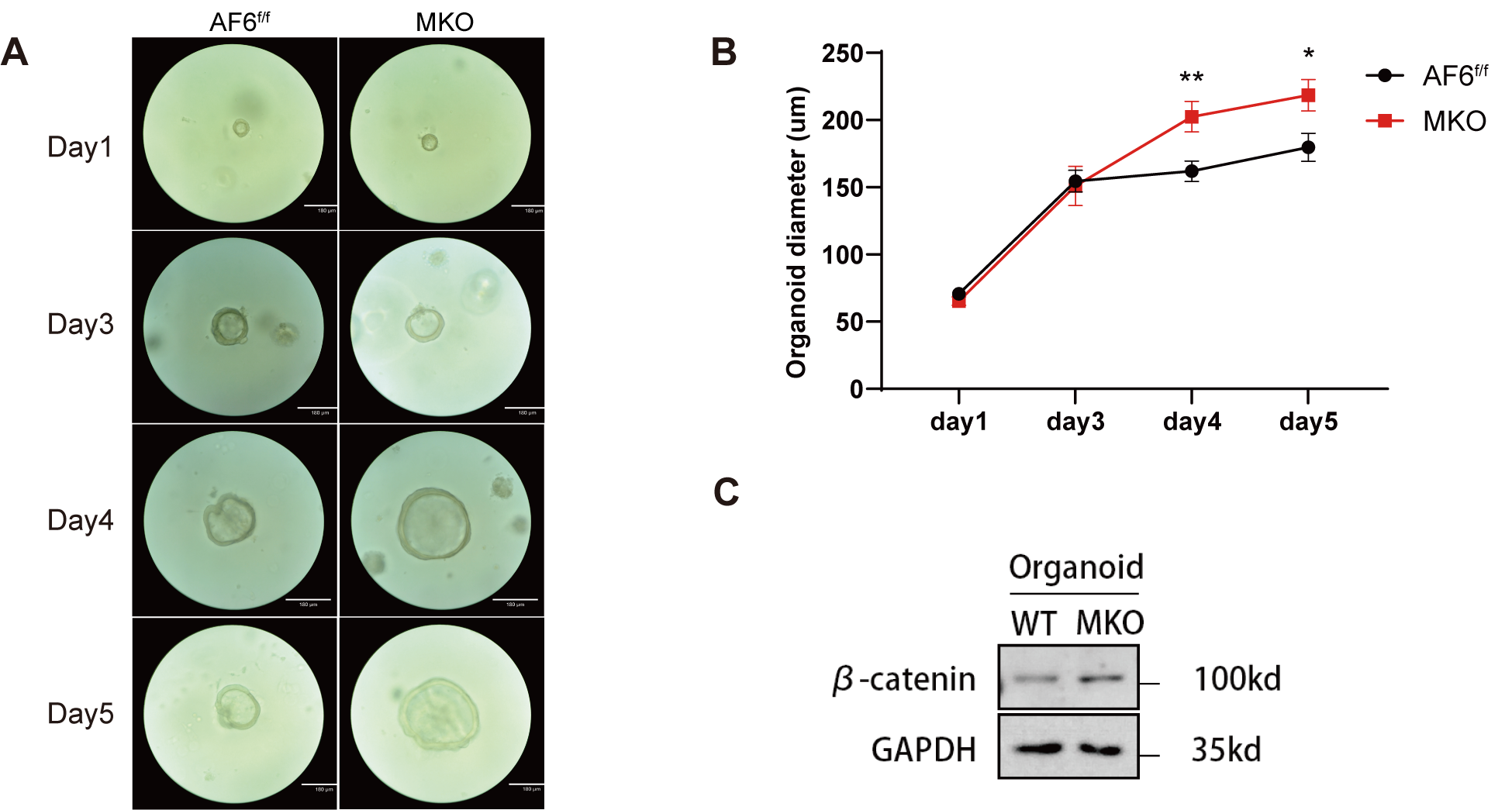

Supplement: Supplementary Figure 1 — AF6 expression is increased in inflammatory macrophages. (A) Expression of AF6 mRNA in various immune cells, which includes bone-marrow-derived macrophages (BMDMs), peritoneal macrophages (PEMs), dendritic cells (MHCII+CD11c+), neutrophils (CD11b+Ly6G+Ly6Clow), T cells (CD3+), and B cells (B220+). The expression levels of AF6 were normalized to the internal control β-actin. (B) UMAP findings illustrate the expression of AF6 in relation to single-cell clusters in macrophages, among mice with colitis and healthy controls. n= 6 per group. (C) UMAP findings illustrate the expression of AF6 in relation to single-cell clusters in macrophages, among patients with CD inflamed (CD), and non-inflamed (CD Health)controls. n= 6 per group. (D) AF6 protein expression was detected by western blot in BMDMs (upper panel) or RAW 264.7 cells (lower panel) treated with LPS or IL-4 for 24h. [file Supplementaryfile1.zip › supp/figure s6.tif]

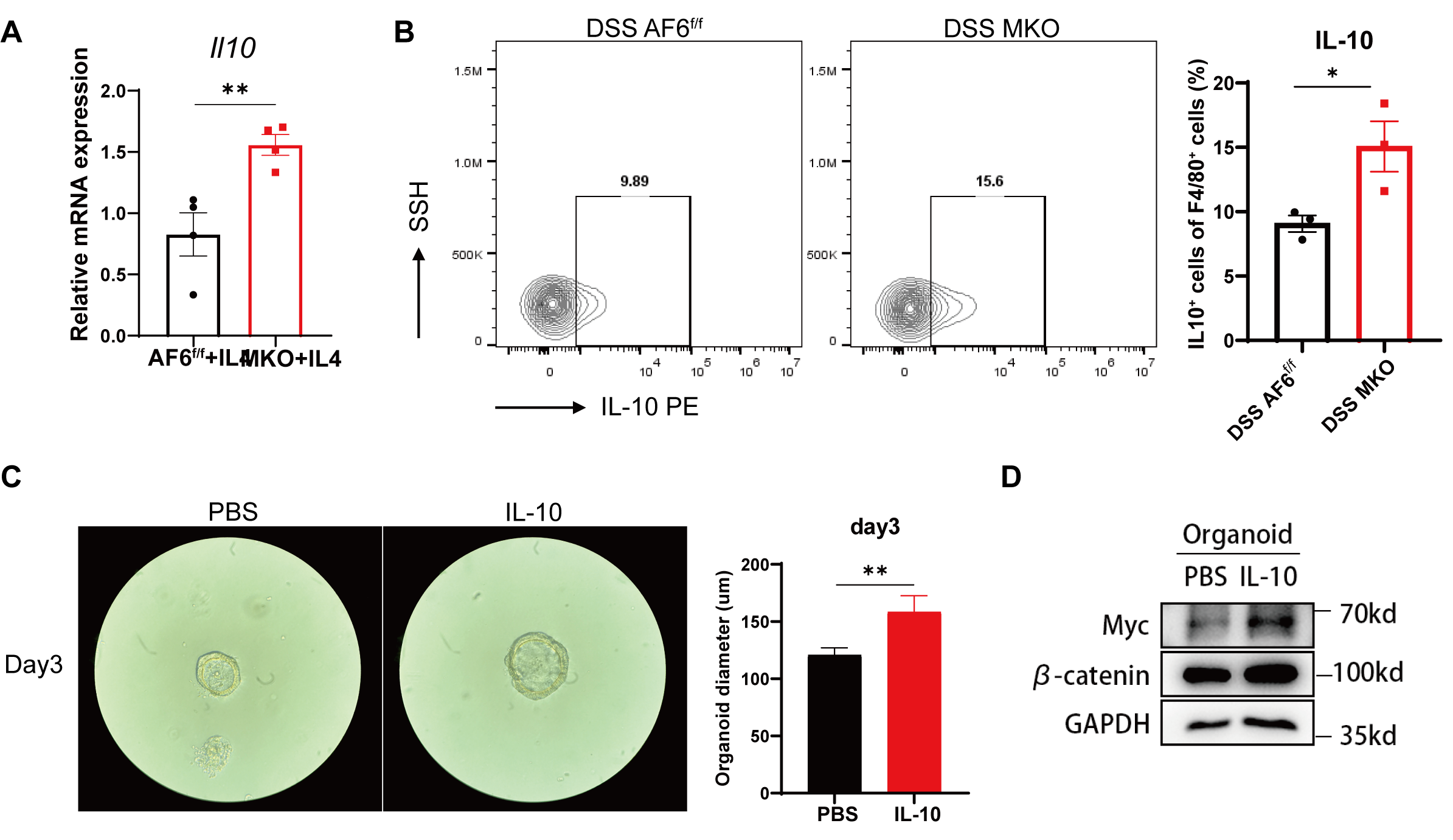

Supplement: Supplementary Figure 1 — AF6 expression is increased in inflammatory macrophages. (A) Expression of AF6 mRNA in various immune cells, which includes bone-marrow-derived macrophages (BMDMs), peritoneal macrophages (PEMs), dendritic cells (MHCII+CD11c+), neutrophils (CD11b+Ly6G+Ly6Clow), T cells (CD3+), and B cells (B220+). The expression levels of AF6 were normalized to the internal control β-actin. (B) UMAP findings illustrate the expression of AF6 in relation to single-cell clusters in macrophages, among mice with colitis and healthy controls. n= 6 per group. (C) UMAP findings illustrate the expression of AF6 in relation to single-cell clusters in macrophages, among patients with CD inflamed (CD), and non-inflamed (CD Health)controls. n= 6 per group. (D) AF6 protein expression was detected by western blot in BMDMs (upper panel) or RAW 264.7 cells (lower panel) treated with LPS or IL-4 for 24h. [file Supplementaryfile1.zip › supp/figure s7.tif]

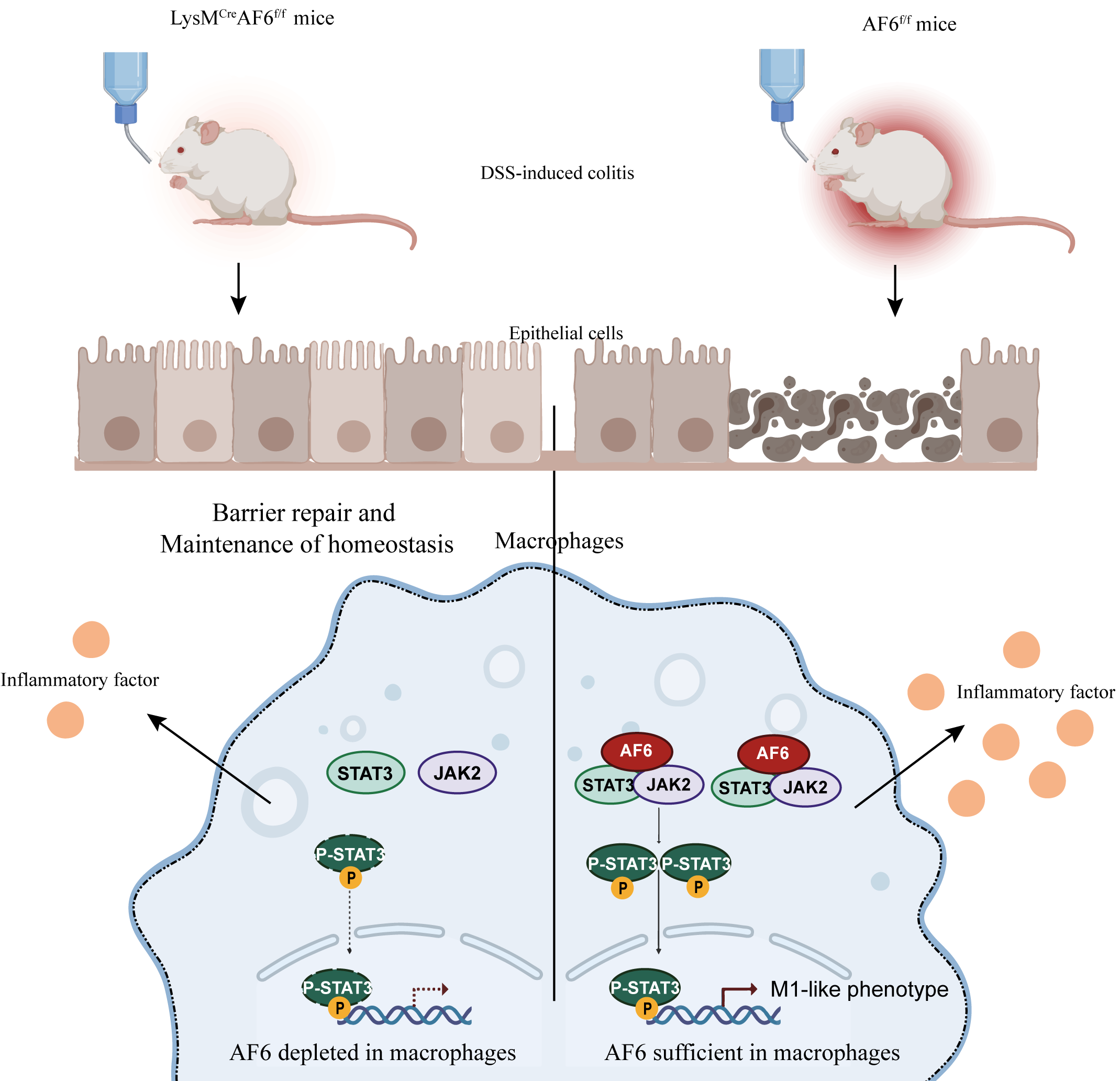

Supplement: Supplementary Figure 1 — AF6 expression is increased in inflammatory macrophages. (A) Expression of AF6 mRNA in various immune cells, which includes bone-marrow-derived macrophages (BMDMs), peritoneal macrophages (PEMs), dendritic cells (MHCII+CD11c+), neutrophils (CD11b+Ly6G+Ly6Clow), T cells (CD3+), and B cells (B220+). The expression levels of AF6 were normalized to the internal control β-actin. (B) UMAP findings illustrate the expression of AF6 in relation to single-cell clusters in macrophages, among mice with colitis and healthy controls. n= 6 per group. (C) UMAP findings illustrate the expression of AF6 in relation to single-cell clusters in macrophages, among patients with CD inflamed (CD), and non-inflamed (CD Health)controls. n= 6 per group. (D) AF6 protein expression was detected by western blot in BMDMs (upper panel) or RAW 264.7 cells (lower panel) treated with LPS or IL-4 for 24h. [file Supplementaryfile1.zip › supp/figure s8.tif]
